# Moral Identity Predicts Adherence to COVID‐19 Mitigation Procedures Depending on Political Ideology: A Comparison Between the USA and New Zealand

**DOI:** 10.1111/pops.12838

**Published:** 2022-06-17

**Authors:** Cillian McHugh, Siobhán M. Griffin, Melanie J. McGrath, Joshua J. Rhee, Paul J. Maher, Darragh McCashin, Jenny Roth

**Affiliations:** ^1^ University of Limerick; ^2^ University of Limerick; ^3^ University of Melbourne; ^4^ University of Melbourne; ^5^ University of Limerick; ^6^ Dublin City University; ^7^ University of Limerick

**Keywords:** COVID‐19, moral iIdentity, polarization, political ideology

## Abstract

Reducing the spread of infectious viruses (e.g., COVID‐19) can depend on societal compliance with effective mitigations. Identifying factors that influence adherence can inform public policy. In many cases, public health messaging has become highly moralized, focusing on the need to act for the greater good. In such contexts, a person's moral identity may influence behavior and serve to increase compliance through different mechanisms: if a person sees compliance as the right thing to do (internalization) and/or if a person perceives compliance as something others will notice as the right thing to do (symbolization). We argue that in societies that are more politically polarized, people's political ideology may interact with their moral identity to predict compliance. We hypothesized that where polarization is high (e.g., USA), moral identity should positively predict compliance for liberals to a greater extent than for conservatives. However, this effect would not occur where polarization is low (e.g., New Zealand). Moral identity, political ideology, and support for three different COVID‐19 mitigation measures were assessed in both nations (*N* = 1,980). Results show that while moral identity can influence compliance, the political context of the nation must also be taken into account.

**Highlights**

- People's moral identity is related to their attitudes toward, and adherence to, COVID‐19 mitigation measures.
- In a context where polarization is high (USA), this relationship is moderated by political ideology; the relationship between moral identity and support for COVID‐19 mitigation measures is observed for liberals, but it is less strong (or even reversed) for conservatives.
- In a context where polarization is lower (New Zealand), this relationship between moral identity and support for COVID‐19 mitigation measures is not moderated by political ideology.
- The results point to the relevance of the national sociopolitical context in shaping how public health messaging advice is received and acted upon, and a consideration of this context may be important in future messaging on societal issues.

In March 2020, the World Health Organization declared a global pandemic due to the spread of severe acute respiratory syndrome coronavirus 2, otherwise known as COVID‐19 (World Health Organization, 2020). Responding to the resulting crisis included large‐scale behavior change, such as engaging in social distancing and changes to hand‐hygiene habits (Patel et al., 2021). However, the effectiveness of these measures to limit the spread of the virus is dependent on compliance from members of society.

Ensuring high levels of adherence to COVID‐19 mitigation measures poses a challenge to policy makers, and compliance varies systematically across nations. We argue that levels of polarization may influence compliance rates across nations. Although public health policy often aims to foster a strong sense of being “in it together” (Foran et al., 2021; Haslam et al., 2018, 2020), even public health goals can become a partisan issue in political contexts where polarization is high (Van Bavel et al., 2020). In addition to these factors, we suggest that moral identity is a key predictor of adherence, especially since public health messages are frequently framed in terms of moral obligations or duties that are necessary to protect others (Grant & Hofmann, 2011; Van Bavel et al., 2020).

The present research examines how moral identity and political ideology influence adherence to COVID‐19 mitigation measures that were set in place by respective governments across different social and cultural contexts. Moral identity refers to a self‐concept organized around a set of moral traits (Aquino & Reed, 2002), and strength of moral identity is a key factor in motivating action aligned with moral values (Erikson, 1964; Hart et al., 1998). We argue that messaging regarding public health responses to COVID‐19 has become highly moralized, and in such a context, people's moral identity is positively associated with adherence to mitigation measures. We further hypothesize that this relationship will be moderated by political ideology in a specific societal climate. In societies where there is social consensus on the moral good of mitigation measures, we predict a positive relationship between moral identity and adherence. However, in societies where mitigation measures are politicized and affective polarization is high, political ideology may itself act as a basis for moral reasoning, interacting with moral identity to affect compliance. We test these predictions by drawing comparisons between two Western cultures with varying levels of polarization and divergent responses to the pandemic: the United States of America and New Zealand.

## COVID‐19: A MORAL CRISIS

As the COVID‐19 pandemic unfolded, it increasingly presented as a moral crisis. In many countries, health care systems became overwhelmed by large numbers of patients, and in some cases, this even led to triage decisions where not everyone could be treated (e.g., Rosenbaum, 2020). Collective responses such as movement restrictions, physical distancing, and increased hygiene mandates were suggested to avoid this stark situation. Therefore, people were asked to work and study from home; schools, shops, and restaurants were closed and in‐person services were not available.

Collective responses of this nature present another set of moral considerations. While these measures may reduce the spread of the virus, they impact different elements of society in unequal ways (e.g., Patel et al., 2020). Not everyone can easily work from home. Many people lost their jobs or were forced to close businesses (at the same time, profits soared for some companies, such as Amazon; see Helmore, 2020). People living alone may disproportionately suffer from the isolation of physical distancing (Fingerman et al., 2021). Policy makers who try to balance these various interests and identify the response that serves the overall greater good are faced with a complex set of ethical dilemmas.

Given these diverse moral considerations, it is no surprise that discourse around the response to the pandemic has become highly moralized (Prosser et al., 2020). Moralization occurs when an issue becomes categorized as morally relevant (McGrath et al., 2019; McHugh et al., 2022) or acquires increased moral significance (Rhee et al., 2019; Rozin, 1999). Moralizing an issue can motivate personal action in relation to that issue (Rhee et al., 2019; van Zomeren, 2013) and generate expectations of others’ behavior (Skitka, 2010; Van Bavel et al., 2020). In the context of COVID‐19, on the one hand, moralization is evident in messaging that highlights public health measures to protect vulnerable members of society as a moral duty—the “right thing to do” (Lewnard & Lo, 2020; Prosser et al., 2020). On the other hand, it is also evident in people's worry about restrictions to people's basic rights or the country's economy.

People vary in what they view as morally right or morally relevant and how much they moralize particular issues (Haidt & Joseph, 2008; Ryan, 2014; Skitka, 2010). Even among those who have moralized a given issue, there is substantial variance in their tendency to allow these moral considerations to motivate their behavior (Aquino et al., 2009). Previous research has suggested moral identity as an indicator of morally motivated behavior (Aquino & Reed, 2002), such that people who score higher in moral identity are more motivated to act in accordance with what they view as moral (e.g., Reynolds & Ceranic, 2007; Shao et al., 2008).

## MORAL IDENTITY AND RESPONSE TO COVID‐19

Moral identity builds on social identity theory (Tajfel & Turner, 1979) to provide a measure of how much people view “being a moral person” as an important aspect of their self‐concept (Aquino & Reed, 2002). Moral identity, as proposed by Aquino and Reed, has two dimensions: *internalization* and *symbolization*. Internalization refers to the importance of moral characteristics to *the self*, whereas symbolization captures the importance of *being seen by others* as a moral person. In the context of COVID‐19, internalization and symbolization may exert distinct influences on behavior. People who score high in internalization will be more likely to adhere to COVID‐19 mitigation measures if they personally perceive this as the right thing to do (Shao et al., 2008), whereas those who score high on symbolization will be more motivated to adhere to guidelines if they perceive that doing so is widely recognized as the right thing to do (Winterich et al., 2013). In the next section, we present ways in which both moral identity dimensions may interact with political ideology to predict compliance with COVID‐19 mitigation measures.

## MORAL IDENTITY AND POLITICAL IDEOLOGY

It is important to acknowledge that various factors can interact with the individual's existing moral values in affecting behavior (Aquino et al., 2009; Boegershausen et al., 2015). Growing evidence suggests that the perceived compatibility of a behavior with the moral content of individuals' political ideology moderates the link between moral identity and behavior (for a review, see Winterich et al., 2015). Indeed, despite the general positive influence of internalization on pro‐social behavior (Reed et al., 2007), high internalizers, compared to low internalizers, have been observed to be *less* generous to charities whose descriptions do not align with the moral foundations of their own political ideology (Winterich et al., 2013) and to display greater moral disengagement toward targets whom they perceived to advance opposing moral values (Gotowiec, 2019). Therefore, evidence suggests that there are situations in which moral identity and individual political ideology may interact to motivate behavior.

Research has demonstrated that people who endorse liberal political ideologies hold different moral values than individuals with more conservative political ideologies (Graham et al., 2009). Typically, liberals prioritize protecting others from harm and treating everyone with fairness, which translates into political attitudes such as opposing the death penalty and medical testing on animals, and supporting gun control and actions to reduce climate change (Graham et al., 2009; Koleva et al., 2012; van Leeuwen & Park, 2009). Liberals also extend this moral consideration to a large circle of people, including friends (rather than just family) and the world (rather than just their own nation; Waytz et al., 2019). This suggests that the moral values of liberals are compatible with public health measures aimed at protecting a wide range of community members from death and illness. As such, morally minded liberals may show greater adherence to the implemented COVID‐19 measures. While conservatives also endorse moral imperatives to avoid suffering and enhance justice, they additionally prioritize a range of other moral values to a greater extent than liberals. These include concerns for loyalty to one's ingroup and respect for tradition and authority (Graham et al., 2009). Furthermore, research on policies addressing climate change mitigation provides evidence that conservatives tend to be morally opposed to mitigation attempts that could interfere with free market principles and the freedom of the individual (Bohr, 2016). In addition, compared to liberals, conservatives have a smaller moral circle, extending their moral consideration more selectively with a preference for family and close ties (Waytz et al., 2019). Taken together, these considerations along with previous findings suggest that motivations to adhere to COVID‐19 measures may conflict with other moral values (e.g., autonomy and freedom) that are more strongly held by conservatives than liberals.

## MORAL IDENTITY AND POLITICAL IDEOLOGY IN DIFFERENT CONTEXTS

We propose that the link between political ideology, moral identity, and COVID‐19 mitigation measures will vary depending on the political context, specifically, the level of affective polarization in the country. Affective polarization refers to a tendency for opposing groups to dislike and distrust each other (e.g., Iyengar et al., 2012). Research has shown that greater affective polarization is associated with more salient political identities and related ingroup norms (e.g., Iyengar & Krupenkin, 2018), which influence attitudes toward political issues (e.g., Bayes et al., 2020). In line with this, we propose that in contexts where affective polarization is high and the response to COVID‐19 has been politicized, political identities are chronically salient such that individuals with a stronger moral identity are more motivated to act in line with their political ideology. Based on the differences between liberal and conservative morality, we expect that liberals with a stronger internalized moral identity will report higher compliance with COVID‐19 measures, whereas highly internalized conservatives will report less compliance. Conversely, where polarization is low and the response to COVID‐19 has not been politicized to the same degree, any association between internalization and individuals' responses to COVID‐19 will not depend on political ideology. Based on the same reasoning, we predict similar effects for symbolization as for internalization. However, acknowledging that these moralization dimensions are conceptually distinct, we test internalization and symbolization separately.

We note that we do not make specific claims about the direction of these relationships, and our hypotheses are consistent with two interpretations of the relationship between moral identity and political ideology. On the one hand, people may have a set of moral values that vary in their relative importance depending on the presence of other identity cues, such as political ideology. On the other hand, moral values may be shaped by political ideology (e.g., through perceived normativity; Lindström et al., 2018), and polarization impacts the range of norms people are exposed to in their socialization (e.g., through ideological sorting; Iyengar & Krupenkin, 2018). Both pathways predict that where polarization is greater, the relationship between moral identity and political ideology in predicting attitudes and behavior should be stronger.^1^

## THE CURRENT RESEARCH

The present research investigated the effect of political ideology on the relationship between moral identity and compliance with COVID‐19 mitigation measures in two national contexts, New Zealand and the USA. Overall, the political system of New Zealand differs from that of the USA on a number of dimensions. For example, New Zealand has only two levels of government, with local councils exercising a limited range of powers conferred by the federal government, rendering it effectively a unitary state. This is in contrast to the USA's bicameral federal legislature and strong federal, state, and local levels of government. Furthermore, while the American head of state is directly elected, in New Zealand the prime minister is drawn from elected parliamentary representatives. The five parties that are currently represented in New Zealand's legislature tend to fit within a liberal–conservative binary. Liberalism and conservatism in New Zealand also manifest in the same pattern of moral foundations endorsement as that observed in the USA, with more conservative individuals expressing stronger support for the binding foundations of the ingroup, authority, and purity, and more liberal people endorsing the individualizing harm and fairness foundations (Davies et al., 2014; Milojev et al., 2014).

Most importantly, these countries differ in levels of affective polarization (Silver et al., 2021), along with the extent to which their response to COVID‐19 has been politicized along ideological lines (e.g., Maher et al., 2022; Moynihan & Roberts, 2021; Vignoles et al., 2021). The USA is experiencing substantial increases in affective polarization compared to New Zealand (Boxell et al., 2020) and is reporting greater incidence of key indicators of affective polarization (Silver et al., 2021). Although evidence is not yet conclusive on the potential effect of electoral systems on affective polarization, some research suggests that interparty animosity is lower in multiparty and proportionally representative systems such as that of New Zealand (Fischer et al., 2021). Furthermore, the response from the respective leaders differed, such that Donald Trump's response was characterized by divisive rhetoric (Moynihan & Roberts, 2021), whereas Jacinda Ardern fostered inclusivity in her response (Vignoles et al., 2021).

In the present study, we examine adherence to three classes of COVID‐19 mitigation measures in each country: support for restrictions (including closure of public venues and schools and restriction of movement), maintenance of physical distance from other people, and adherence to physical hygiene measures such as frequent handwashing. We present four hypotheses regarding the role of moral identity in predicting support for COVID‐19 mitigation measures. Although theory predicts similar effects for both internalization and symbolization components of moral identity, we anticipate the possibility of diverging influences. For example, if there is ambiguity regarding what is *seen as the right thing to do*, the role of symbolization will be less clear. As such, we present and test our hypotheses for internalization and symbolization separately.

We hypothesize that moral identity will predict greater adherence to COVID‐19 mitigation measures (H1), and this will be observed for both internalization (H1a) and symbolization (H1b). Additionally, we predict that the relationship between moral identity and adherence is moderated by country and political ideology (H2), again for both internalization (H2a) and symbolization (H2b). Specifically, for the USA, where polarization is high, we predict an interaction between moral identity and political ideology (H3; for internalization [H3a] and symbolization [H3b]), whereby for liberals, moral identity predicts greater adherence to COVID‐19 measures, while for conservatives, this relationship is less pronounced (and may even be reversed—especially for restrictions relating to economic activity and the closure of businesses). In contrast, for New Zealand, we predict this interaction to be significantly weaker (H4; for both internalization [H4a] and symbolization [H4b]) due to the lower levels of polarization in New Zealand.

In our analyses, we control for risk perception, narcissism, belief in conspiracy theories, and national identification to investigate the predicted effects independent of variables that likely influence adherence to COVID‐19 mitigation measures. We anticipate that people who perceive a higher risk from COVID‐19 will be more likely to adhere to mitigation measures. Individual‐level narcissism has been previously linked with health‐related behaviors (e.g., Dębska et al., 2021), and collective narcissism (Golec de Zavala et al., 2009) may lead to a decreased focus on mitigation strategies in favor of defending a country's image (Van Bavel et al., 2022). Furthermore, belief in conspiracy theories may undermine adherence to mitigation strategies (Earnshaw et al., 2020), while national identification has been linked with adherence to mitigation strategies (Van Bavel et al., 2022).

Our hypotheses were preregistered at https://aspredicted.org/CNK_SNP prior to gaining access to the full dataset. We note three deviations from the preregistration. First, here we provide greater detail and specificity compared to the preregistration. Second, in the preregistration, we did not distinguish between internalization and symbolization. Although the theory predicts similar effects for both, we anticipate the possibility of diverging influences (e.g., the role of symbolization can be less clear). Third, in the preregistration, we predicted similar effects for political ideology and moral circle; however, here we focus on political ideology. The primary rationale for this decision is to reduce complexity and allow more in‐depth discussion of political ideology, which has emerged as an important consideration in responses to COVID‐19 (e.g., Ruisch et al., 2021). Moral circle was included in all analyses, and the results involving moral circle are reported in the supplementary materials, though we note the majority of the registered predicted interactions were not observed.

## METHOD

### Participants and Procedure

In April 2020, as part of a large‐scale international project, data were collected from 46,450 participants in 67 countries (22,160 females, 23,948 males, *M*_age_ = 43.09, *SD* = 16.22).^2^ In the present research, we focus on data from the New Zealand and the USA samples (*N* = 1,980) due to the differences in polarization (New Zealand, *n* = 509; USA, *n* = 1,471). Participants were representative with respect to age (*M*_age_ = 44.70, *SD* = 16.76, range = 18–89) and gender (965 females, 1,008 males, 5 other, 2 missing); full participant data are presented in Table S1. At this stage of the pandemic, New Zealand reported 301.02 cases per million in the population and 3.89 deaths per million, whereas the USA reported 3,010.96 cases per million in the population and 171.42 deaths per million. Data were collected via an online survey presented on the Lucid survey sampling platform. Participants completed a series of psychometric scales and self‐reported their support for various COVID‐19 mitigation measures, all of which were presented in random order. The internal consistency of all scales reported here was above the minimum acceptable limit of α = .7 as proposed by Field et al. (2012).

### Materials

#### COVID‐19 Mitigation Measures

We assessed three types of mitigation measures: support for restrictions (henceforth restrictions support), adherence to physical distancing measures (henceforth distancing adherence), and adherence to physical hygiene measures (henceforth hygiene adherence). All items were rated on a slider scale, ranging from 0 = *strongly disagree* to 100 = *strongly agree*, with 11 anchors (0, 10, 20, etc.); thus, higher scores indicate stronger support and greater adherence (full wordings of all items are in the online appendix).

##### Restrictions support

Five items assessed support for policy restrictions (e.g., “In favor of closing all bars and restaurants”; Cronbach's α: total sample: .91, USA: .91, New Zealand: .88).

##### Distancing adherence

Five items assessed adherence to physical distancing measures (e.g., “Keeping physical distance from all other people outside my home”; Cronbach's α: total sample: .77, USA: .76, New Zealand: .77).

##### Hygiene adherence

Five items assessed adherence to physical hygiene guidelines (e.g., “Washing my hands immediately after returning home”; Cronbach's α: total sample: .85, USA: .87, New Zealand: .80).

#### Moral Identity

The 10‐item Moral Identity Scale (Aquino & Reed, 2002) was employed. Five items assessed symbolization (e.g., “I am actively involved in activities that communicate to others that I have these characteristics”; Cronbach's α: total sample: .835, USA: .84, New Zealand: .80), and five items assessed internalization (e.g., “Being someone who has these characteristics is an important part of who I am”; Cronbach's α: total sample: .73, USA: .73, New Zealand: .77). Responses were recorded on an 11‐point scale ranging from 0 = *strongly disagree* to 10 = *strongly agree*; negative items were reverse‐coded such that higher scores indicated higher moral identity.

#### Political Ideology

We measured participants' political ideology using a left–right self‐placement item (“Overall, how would you describe yourself in terms of political ideology?”), where participants indicated their orientation as ranging from 0 = *Extremely liberal/left leaning* to 10 = *Extremely conservative/very right leaning*. This widely used measure is generally stable (Krosnick, 1991) and predicts various social attitudes (Dunn, 2011).

#### Covariates

##### Moral circle

The moral circle task (Waytz et al., 2019) was employed, whereby participants indicate how far their moral circle in general extends (i.e., categories for whom a person is concerned about right and wrong done to them) by selecting 1 of 16 statements ranging from *your immediate family* to *all things in existence*. Moral circle is thus scored from 1 to 16, where larger numbers represent a larger moral circle (see the online appendix for full wording).

##### Risk perception

COVID‐19 risk perception was assessed by two items: “By April 30, 2021: How likely do you think it is that you will get infected by the Coronavirus (Covid‐19)?” and “By April 30, 2021: How likely do you think it is that the average person in [your country] will get infected by the Coronavirus (COVID‐19)?” Each item was rated from 0% = *impossible* to 100% = *certain*. Higher scores represent higher perceived risk (Cronbach's α: total sample: .84, USA: .82, New Zealand: .87).

##### Individual narcissism

The six‐item Brief Narcissistic Admiration and Rivalry Questionnaire (NARQ; Back et al., 2013) assessed two dimensions of narcissism: admiration (e.g., “I deserve to be seen as a great personality”; Cronbach's α: combined sample: .83, USA: .83, New Zealand: .79) and rivalry (e.g., “Most people are somehow losers”; Cronbach's α: total sample: .81, USA: .83, New Zealand: .73). Responses were recorded on an 11‐point scale ranging from 0 = *strongly disagree* to 10 = *strongly agree*, with higher scores representing higher admiration and rivalry.

##### Collective narcissism

Collective narcissism was assessed using a three‐item scale (Golec de Zavala et al., 2009; e.g., “[My national group] deserves special treatment”). Responses were recorded on an 11‐point scale ranging from 0 = *strongly disagree* to 10 = *strongly agree*. Higher scores represent greater collective narcissism (Cronbach's α: total sample: .85, USA: .86, New Zealand: .80).

##### Conspiracy beliefs

Four items assessed COVID‐19 conspiracy beliefs (e.g., “The coronavirus (COVID‐19) is a bioweapon engineered by scientists.” Participants rated each item from 0 = *strongly disagree* to 10 = *strongly agree*. Higher scores represent stronger conspiracy beliefs (Cronbach's α: total sample: .92, USA: .92, New Zealand: .92).

##### National identification

Two items assessed national identification (“I identify as [nationality]”; Postmes et al., 2013; “Being a [nationality] is an important reflection of who I am”; Leach et al., 2008). Responses were recorded on an 11‐item scale ranging from 0 = *strongly disagree* to 10 = *strongly agree* (Cronbach's α: total sample: .77, USA: .76, New Zealand: .80). Higher scores indicate higher levels of national identification.

## RESULTS

In the analysis that follows, effects for the variables of interest (moral identity internalization and symbolization, political ideology, and country) are reported; these values are from the full model including the above specified covariates. All the main analyses are conducted on mean‐centered data. To aid in interpretation, we report raw (non‐mean‐centered) scores for all variables in Tables 1 and 4, and in all figures.

**Table 1:** Bivariate Correlations for Total Sample (USA and New Zealand), with Means and Standard Devations)

	1	2	3	4	5	*M*	*SD*
1. Restrictions support	–					7.82	2.30
2. Distancing adherence	.55**	–				8.13	1.77
3. Hygiene adherence	.41**	.39**	–			7.86	1.90
4. Internalization	.30**	.53**	.27**	–		7.44	1.86
5. Symbolization	.16**	−.03	.33**	−.02	–	5.89	2.10
6. Political ideology	−.10**	−.15**	.12**	−.16**	.31**	5.89	2.58

* *p* < .05;

** *p* < .001.

**Table 2:** Bivariate Correlations for the USA Subsample

	1	2	3	4	5
1. Restrictions support	–				
2. Distancing adherence	.54**	–			
3. Hygiene adherence	.48**	.50**	–		
4. Internalization	.28**	.55**	.31**	–	
5. Symbolization	.23**	.03	.33**	−.02	–
6. Political ideology	−.07*	−.12**	.14**	−.14**	.33**

* *p* < .05;

** *p* < .001.

For zero‐order correlations between the variables of interest, see Tables 1, 2, 3. Full statistics, including the covariates, are reported in the online appendix (correlations: Tables S2–S4, *t*‐test: Table S5, regression analyses: Tables S7–S18).

**Table 3:** Bivariate Correlations for the New Zealand Subsample

	1	2	3	4	5
1. Restrictions support	–				
2. Distancing adherence	.50**	–			
3. Hygiene adherence	.28**	.20**	–		
5. Internalization	.33**	.45**	.18**	–	
6. Symbolization	.05	−.10*	.30**	.04	–
7. Political ideology	−.10*	−.11*	−.01	−.22**	.14**

* sig. at *p* < .05;

** sig. at *p* < .001.

To test for differences in these variables between the two subsamples, we conducted a series of *t‐*tests. Participants in the New Zealand subsample reported greater restrictions support, greater distancing adherence, and greater hygiene adherence, and they scored higher on internalization than participants in the USA subsample. Participants in the USA subsample only scored significantly higher on symbolization than participants in the New Zealand subsample; results are reported in Table 4 (for results including covariates, see Table S5; see Table S6 for gender differences).

**Table 4:** Means, Standard Deviations, and Differences Between the Subsamples

	USA		NZL		*t*	*df*	*p*	*d*
	*M*	*SD*	*M*	*SD*				
Restrictions support	7.58	2.39	8.53	1.84	9.22	1138.13	<.001**	0.42
Distancing adherence	7.89	1.81	8.84	1.45	11.85	1092.8	<.001**	0.55
Hygiene adherence	7.99	1.90	7.5	1.85	−5.11	903.28	<.001**	0.26
Internalization	7.37	1.91	7.64	1.7	3.02	982.03	.003*	0.15
Symbolization	6.07	2.13	5.37	1.91	−6.84	980.43	<.001**	0.33
Political ideology	6.11	2.69	5.23	2.12	−7.53	1105.91	<.001**	0.35

*Note*: NZL, New Zealand.

* *p* < .05;

** *p* < .001.

### Responses to COVID‐19 Measures Depending on Moral Identity, Political Ideology, and Country

Below, we present the tests of our hypotheses for each dependent measure. We report the results for the overall model and any relevant relationships for the variables of interest. All models are numbered and displayed in full in the online appendix. Table 5 presents an overview of our hypotheses.

**Table 5:** Summary of Hypotheses

	Expected Relationship
H1a	Higher internalization scores predict greater adherence to mitigation measures
H1b	Higher symbolization scores predict greater adherence to mitigation measures
H2a	A three‐way Country × Internalization × Political Ideology interaction predicting adherence
H2b	A three‐way Country × Symbolization × Political Ideology interaction predicting adherence
H3a	An Internalization × Political Ideology interaction predicting adherence in the USA
H3b	A Symbolization × Political Ideology interaction predicting adherence in the USA
H4a	Weaker/No Internalization × Political Ideology interaction predicting adherence in New Zealand
H4b	Weaker/No Symbolization × Political Ideology interaction predicting adherence in New Zealand

#### H1a: Internalization and Adherence

First, we conducted regression models including all variables across the full dataset (without accounting for country) for each of our outcome measures (restrictions support, distancing adherence, and hygiene adherence). All models were significant: restrictions support, *R* ^2^ = 0.25, *SE* = 0.87, adjusted *R* ^2^ = 0.24, *F*(17, 1932) = 37.28, *p* < .001 (Table S7, Model 1); distancing adherence, *R* ^2^ = 0.36, *SE* = 0.8, adjusted *R* ^2^ = 0.36, *F*(17, 1932) = 64.68, *p* < .001 (Table S11, Model 5); and hygiene adherence, *R* ^2^ = 0.27, *SE* = 0.86, adjusted *R* ^2^ = 0.26, *F*(17, 1931) = 41.61, *p* < .001 (Table S15, Model 9). For each model, internalization significantly predicted all three outcome measures: restrictions support, *b* = 0.22, *SE* = 0.03, 95% CI [0.16, 0.27], *t*(1932) = 8.29, *p* < .001; distancing adherence, *b* = 0.34, *SE* = 0.02, 95% CI [0.29, 0.38], *t*(1932) = 14.02, *p* < .001; and hygiene adherence, *b* = 0.20, *SE* = 0.03, 95% CI [0.15, 0.25], *t*(1931) = 7.82, *p* < .001. Thus, we found support for the hypothesis that internalization would predict greater adherence to COVID‐19 mitigation measures (H1a).

#### H1b: Symbolization and Adherence

We report the results for symbolization from the same models described above; as such, we avoid repetition and do not re‐report the overall models. Symbolization significantly predicted restriction support, *b* = 0.14, *SE* = 0.02, 95% CI [0.10, 0.19], *t*(1932) = 5.88, *p* < .001 (Table S7, Model 1); distancing adherence, *b* = 0.05, *SE* = 0.02, 95% CI [0.01, 0.10], *t*(1932) = 2.37, *p* = .018 (Table S11, Model 5); and hygiene adherence, *b* = 0.23, *SE* = 0.02, 95% CI [0.18, 0.28], *t*(1931) = 9.44, *p* < .001 (Table S15, Model 9), supporting H1b that symbolization would predict greater adherence to COVID‐19 mitigation measures.

#### H2a: Internalization × Political Ideology × Country Interaction

To test the predicted three‐way interactions proposed between internalization, political ideology, and country (H2), we added country as a variable in our regression models. All three models remained significant and performed significantly better with the inclusion of country, significantly predicting restrictions support, *R* ^2^ = 0.30, *SE* = 0.84, adjusted *R* ^2^ = 0.29, *F*(35, 1914) = 23.67, *p* < .001 (Table S8, Model 2; *F*_*change*_[18, 1914] = 8.38, *p* < .001); distancing adherence, *R* ^2^ = 0.42, *SE* = 0.77, adjusted *R* ^2^ = 0.41, *F*(35, 1914) = 38.94, *p* < .001 (Table S12, Model 6; *F*_*change*_[18, 1914] = 9.68, *p* < .001); and hygiene adherence, *R* ^2^ = 0.28, *SE* = 0.85, adjusted *R* ^2^ = 0.27, *F*(35, 1913) = 21.76, *p* < .001 (Table S16, Model 10; *F*_*change*_[18, 1913] = 2.47, *p* < .001).

We found a significant three‐way Internalization × Political Ideology × Country interaction predicting restrictions support, *b* = −0.15, *SE* = 0.06, 95% CI [−0.26, −0.04], *t*(1914) = −2.69, *p* = .007 (Table S8, Model 2), and distancing adherence, *b* = −0.11, *SE* = 0.05, 95% CI [−0.21, −0.01], *t*(1932) = −2.06, *p* = .039 (Table S12, Model 6). This three‐way interaction was not observed for hygiene adherence, *b* = 0.06, 95% CI [−0.06, 0.17], *t*(1913) = 0.98, *p* = .327 (Table S16, Model 10). Together, these results provide some support for H2a: Greater internalization predicted greater restrictions support and greater distancing adherence, and this was moderated by political ideology in the USA but not New Zealand. We describe this in more detail below when discussing the countries separately. Briefly, in the USA the relationship between internalization and distancing adherence was stronger for liberals than conservatives; for restrictions support, internalization predicted greater support in the case of liberals and moderates, but predicted *less* support in the case of conservatives. In the New Zealand sample, these interactions with political ideology were not observed.

#### H2b: Symbolization × Political Ideology × Country Interaction

We did not find similar results for symbolization. The predicted three‐way Country × Symbolization × Political Ideology interaction was not observed for restrictions support, *b* = 0.06, *SE* = 0.05, 95% CI [−0.03, 0.16], *t*(1914) = 1.28, *p* = .202 (Table S8, Model 2); distancing adherence, *b* = 0.06, *SE* = 0.05, 95% CI [−0.03, 0.15], *t*(1932) = 1.26, *p* = .209 (Table S12, Model 6); or hygiene adherence, *b* = 0.04, 95% CI [−0.06, 0.14], *t*(1913) = 0.71, *p* = .476 (Table S16, Model 10), failing to support H2b.

#### H3a: Internalization and Political Ideology in the USA Sample

We ran separate regression analyses on the USA and New Zealand samples to further investigate the relationships observed.

##### Restrictions support

**Figure 1:**
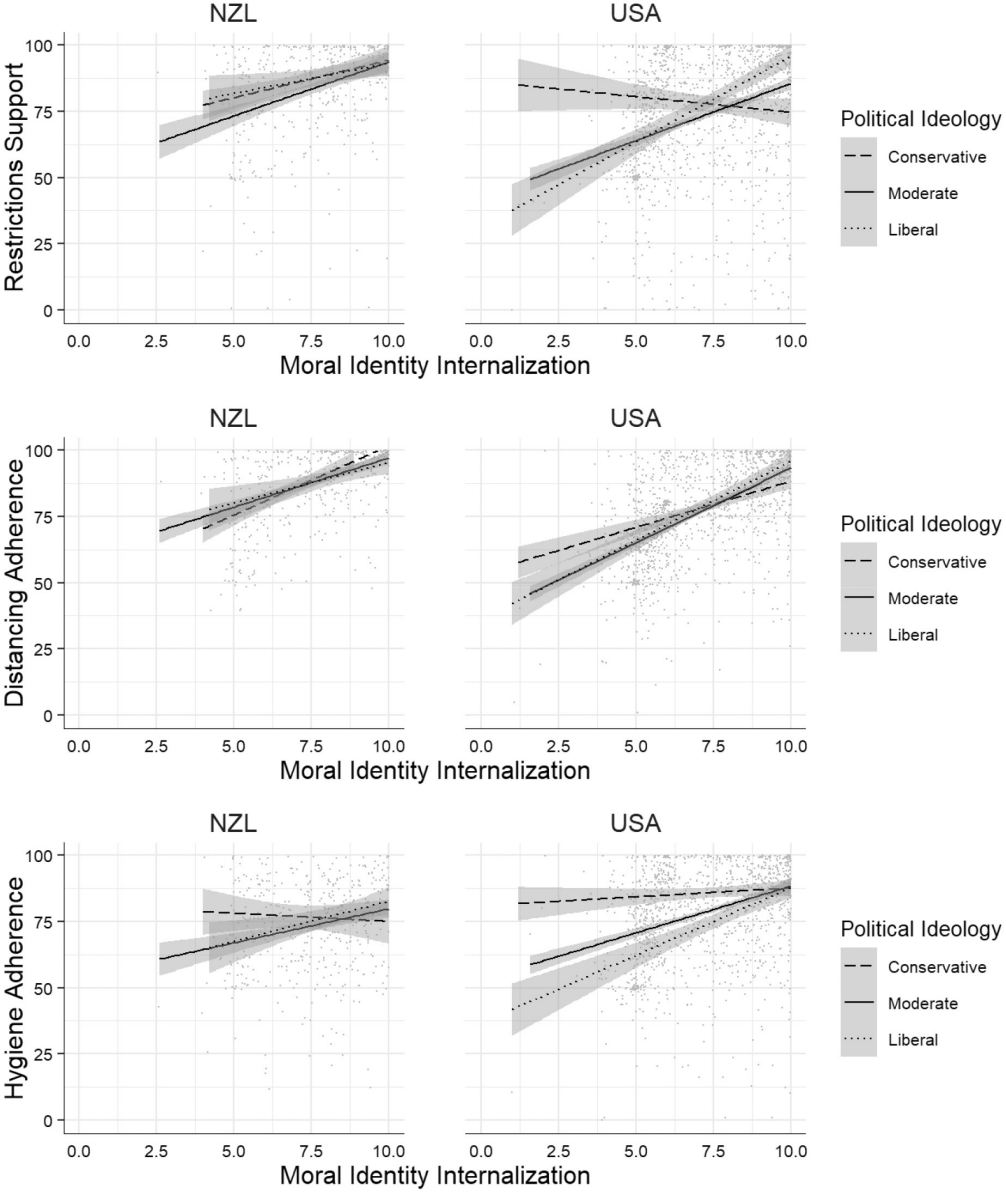
Political Ideology × Internalization interaction for each dependent measure depending on country.

First, we conducted a regression for all variables across the USA sample, with restrictions support as the outcome measure. This model was significant, *R* ^2^ = 0.31, *SE* = 0.83, adjusted *R* ^2^ = 0.3, *F*(17, 1429) = 37.46, *p* < .001 (Table S9, Model 3). Political ideology, *b* = −0.14, *SE* = 0.03, 95% CI [−0.19, −0.08], *t*(1430) = −4.83, *p* < .001, and internalization, *b* = 0.22, *SE* = 0.03, 95% CI [0.16, 0.28], *t*(1430) = 7.26, *p* < .001, significantly predicted restrictions support. As predicted, we found a significant Internalization × Political Ideology interaction, *b* = −0.12, *SE* = 0.02, 95% CI [−0.17, −0.08], *t*(1430) = −5.46, *p* < .001. Greater internalization predicted greater restrictions support in the case of liberals and moderates, but it predicted less support in the case of conservatives (see Figure 1).

##### Distancing adherence

The same model with distancing adherence as the outcome measure, was also significant, *R* ^2^ = 0.4, *SE* = 0.8, adjusted *R* ^2^ = 0.39, *F*(17, 1430) = 55.3, *p* < .001, (Model 7, Table S13). Internalization significantly predicted distancing adherence, *b* = 0.35, *SE* = 0.03, 95% CI = [0.30, 0.41], *t*(1430) = 12.66, *p* < .001, while political ideology did not, *b* = −0.02, *SE* = 0.03, 95% CI = [−0.07, 0.03], *t*(1430) = −0.87, *p* = .387. The predicted Internalization × Political Ideology interaction was observed, *b* = −0.05, *SE* = 0.02, 95% CI = [−0.09, −0.01], *t*(1430) = −2.59, *p* = .010. Greater internalization predicted greater distancing adherence, and this effect was stronger for liberals and moderates than for conservatives (see Figure 1).

##### Hygiene adherence

Finally, the same model with hygiene adherence as the outcome measure was also significant, *R* ^2^ = 0.31, *SE* = 0.83, adjusted *R* ^2^ = 0.3, *F*(17, 1429) = 37.46, *p* < .001, (Model 11, Table S17). Internalization significantly predicted hygiene adherence, *b* = 0.22, *SE* = 0.03, 95% CI = [0.16, 0.28], *t*(1429) = 7.58, *p* < .001, as did political ideology, *b* = 0.22, *SE* = 0.03, 95% CI = [0.16, 0.28], *t*(1429) = 7.58, *p* < .001. The predicted Internalization × Political Ideology interaction was observed, *b* = −0.06, *SE* = 0.02, 95% CI = [−0.11, −0.02], *t*(1429) = −2.95, *p* = .003. Greater internalization predicted greater hygiene adherence for liberals and moderates, while conservatives showed strong adherence to physical hygiene measures regardless of internalization (see Figure 1).

In sum, in the USA sample, the predicted Internalization × Political Ideology interactions were observed for all three mitigation measures, supporting H3a.

#### H3b: Symbolization and Political Ideology in the USA Sample

The results below are taken from the same models described above.

##### Restrictions support

**Figure 2:**
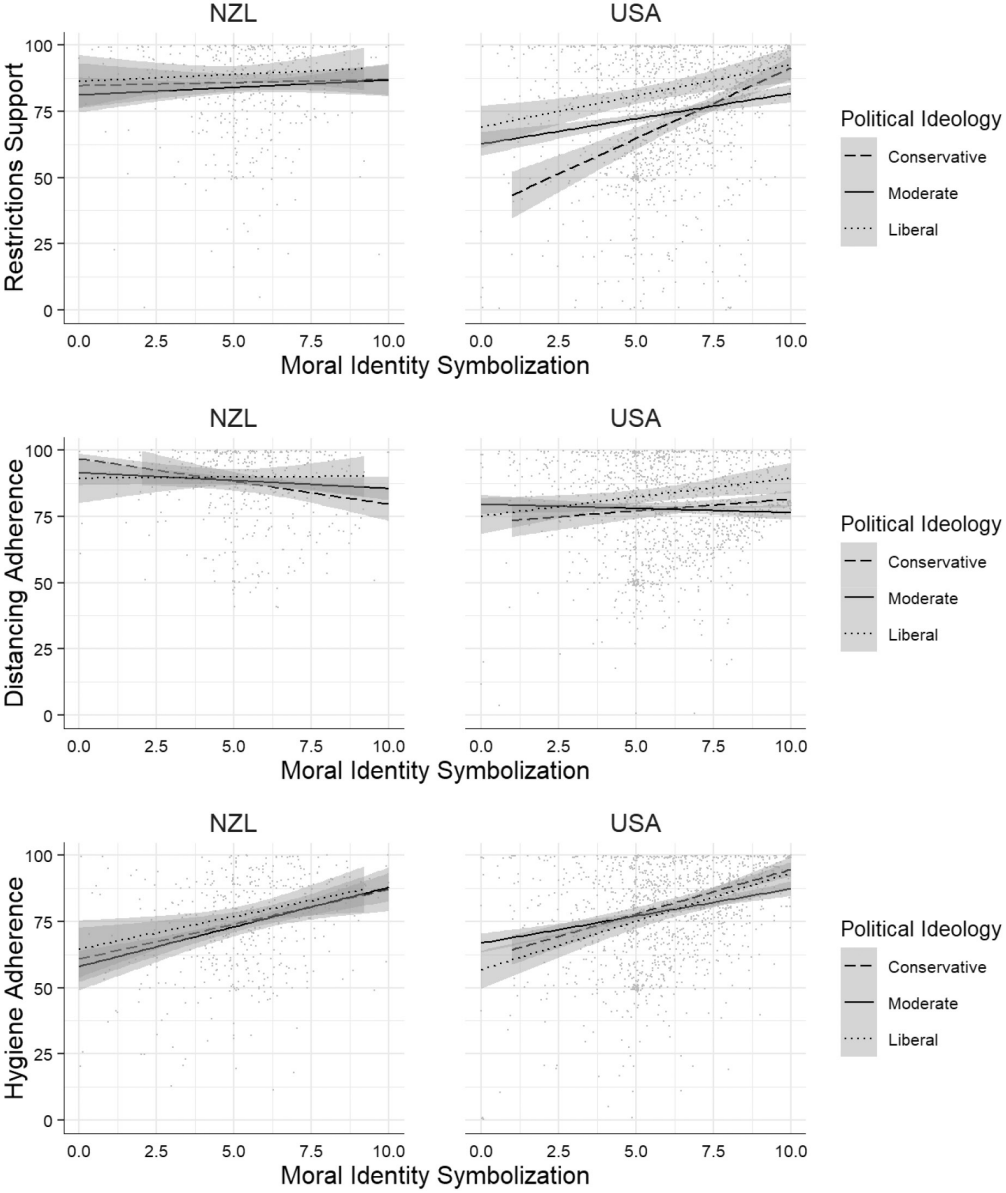
Political Ideology × Symbolization interaction for each dependent measure depending on country.

In addition to internalization and political ideology, symbolization also significantly predicted restrictions support, *b* = 0.18, *SE* = 0.03, 95% CI [0.13, 0.24], *t*(1430) = 6.37, *p* < .001, (Table S9, Model 3). The Symbolization × Political Ideology interaction was significant, *b* = 0.11, *SE* = 0.02, 95% CI [0.07, 0.15], *t*(1430) = 5.47, *p* < .001. Greater symbolization predicted greater restrictions support. Interestingly, this relationship appears to be stronger for conservatives than for liberals and moderates (see Figure 2).

##### Distancing adherence

For distancing adherence, symbolization was also a significant predictor in the model, *b* = 0.10, *SE* = 0.03, 95% CI [0.05, 0.16], *t*(1430) = 3.99, *p* < .001 (Table S13, Model 7). The predicted Symbolization × Political Ideology interaction was significant, *b* = 0.07, *SE* = 0.02, 95% CI [0.04, 0.11], *t*(1430) = 3.85, *p* < .001. Greater symbolization predicted greater adherence to physical distancing measures for liberals, and to a lesser extent conservatives, but not for moderates (see Figure 2).

##### Hygiene adherence

Symbolization significantly predicted hygiene adherence, *b* = 0.22, *SE* = 0.03, 95% CI [0.16, 0.27], *t*(1429) = 7.84, *p* < .001 (Table S17, Model 11). The predicted Symbolization × Political Ideology interaction was not present, *b* = 0.03, *SE* = 0.02, 95% CI [−0.01, 0.07], *t*(1429) = 1.62, *p* = .106 (see Figure 2).

In sum, in the USA sample, a significant Symbolization × Political Ideology interaction was observed for restrictions support and distancing adherence, but not hygiene adherence, providing some support for H3b.

#### H4a: Internalization and Political Ideology in the New Zealand Sample

##### Restrictions support

First, we conducted regressions for all variables across the New Zealand sample, with restrictions support as the outcome measure. This model was significant, *R* ^2^ = 0.21, *SE* = 0.72, adjusted *R* ^2^ = 0.18, *F*(17, 484) = 7.52, *p* < .001 (Table S10, Model 4). Internalization was a significant predictor, *b* = 0.21, *SE* = 0.04, 95% CI [0.12, 0.29], *t*(484) = 4.63, *p* < .001; political ideology was not, *b* = −0.02, *SE* = 0.04, 95% CI [−0.10, 0.07], *t*(484) = −0.34, *p* = .732. There was no significant Internalization × Political Ideology interaction, *b* = 0.03, *SE* = 0.04, 95% CI [−0.06, 0.11], *t*(484) = 0.60, *p* = .550.

##### Distancing adherence

The same regression, but with distancing adherence as the outcome measure, was also significant, *R* ^2^ = 0.32, *SE* = 0.69, adjusted *R* ^2^ = 0.3, *F*(17, 484) = 13.4, *p* < .001 (Table S14, Model 8). Internalization significantly predicted distancing adherence, *b* = 0.27, *SE* = 0.04, 95% CI [0.19, 0.35], *t*(484) = 6.32, *p* < .001, whereas political ideology did not, *b* = 0.06, *SE* = 0.04, 95% CI [−0.02, 0.14], *t*(484) = 1.45, *p* = .148. Again, there was no significant Internalization × Political Ideology interaction, *b* = 0.05, *SE* = 0.04, 95% CI [−0.03, 0.14], *t*(484) = 1.25, *p* = .213.

##### Hygiene adherence

Finally, the same regression for hygiene adherence as the outcome measure was significant, *R* ^2^ = 0.17, *SE* = 0.9, adjusted *R* ^2^ = 0.14, *F*(17, 484) = 5.93, *p* < .001 (Table S18, Model 12). Internalization did not significantly predict adherence, *b* = 0.09, *SE* = 0.06, 95% CI [−0.02, 0.20], *t*(484) = 1.68, *p* = .093, nor did political ideology, *b* = 0.02, *SE* = 0.06, 95% CI [−0.09, 0.13], *t*(484) = 0.34, *p* = .734. Interestingly we found a significant Internalization × Political Ideology interaction, *b* = −0.12, *SE* = 0.05, 95% CI [−0.23, −0.01], *t*(484) = −2.15, *p* = .032. Greater internalization predicted greater hygiene adherence for liberals and moderates, but not for conservatives (see Figure 1).

In sum, in line with our predictions, in the New Zealand sample, there was no Internalization × Political Ideology interaction for distancing adherence or restrictions support, though we did find this interaction for hygiene adherence (see Figure 1). Thus, we found some support for H4a (that this interaction would be weak in New Zealand due to low levels of polarization).

#### H4b: Symbolization and Political Ideology in the New Zealand Sample

The results below are taken from the same models described above.

##### Restrictions support

In the New Zealand sample, symbolization did not predict restrictions support, *b* = 0.06, *SE* = 0.04, 95% CI [−0.03, 0.14], *t*(484) = 1.29, *p* = .199 (Table S10, Model 4). There was no Symbolization × Political Ideology interaction, *b* = 0.05, *SE* = 0.04, 95% CI [−0.03, 0.13], *t*(484) = 1.19, *p* = .233.

##### Distancing adherence

For distancing adherence, symbolization was not a significant predictor in the model, *b* = −0.07, *SE* = 0.04, 95% CI [−0.15, 0.01], *t*(484) = −1.73, *p* = .085 (Table S14, Model 8); there was no significant Symbolization × Political Ideology interaction, *b* = 0.01, *SE* = 0.04, 95% CI [−0.06, 0.09], *t*(484) = 0.35, *p* = .726.

##### Hygiene adherence

Symbolization significantly predicted hygiene adherence, *b* = 0.22, *SE* = 0.05, 95% CI [0.12, 0.33], *t*(484) = 4.12, *p* < .001 (Table S18, Model 12). There was no significant Symbolization × Political Ideology interaction, *b* = −0.01, *SE* = 0.05, 95% CI [−0.10, 0.09], *t*(484) = −0.10, *p* = .919.

In sum, in line with our predictions, in the New Zealand sample there was no Symbolization × Political Ideology interaction on any of the mitigation measures (see Figure 2), supporting H4b (that this interaction would be weak in New Zealand due to low levels of polarization).

### Exploratory Analyses: Opposition to Mitigation Measures

Above, we presented results for moral identity, political ideology, and country on their effects for support for various mitigation measures. We did not distinguish between lower levels of support and overt opposition or defiance toward the mitigation measures. Given that discourse surrounding mitigation measures has become characterized by overt opposition (e.g., anti‐vax/anti‐mask rhetoric; see Altiparmakis et al., 2021; Earnshaw et al., 2020) we conducted a follow‐up analysis to explore this specifically. We recoded our outcome measures to binary variables. Responses on or above the midpoint were coded as indicating agreement with the mitigation measures, and responses below the midpoint coded as disagreement with the measures (interpreted as opposition/defiance).

Overall, rates of defiance were low, with 191 participants (9.69%) opposing restrictions according to the cut‐off employed (USA: *N* = 172, 11.74%; New Zealand: *N* = 19, 3.75%), 84 participants (4.26%) opposing physical distancing measures (USA: *N* = 75, 5.12%; New Zealand: *N* = 9, 1.78%), and 113 participants (5.73%) opposing hygiene measures (USA: *N* = 77, 5.26%; New Zealand: *N* = 36, 7.11%). We conducted a series of logistic regressions to test the predictors of defiance for each mitigation measure (see online appendix Tables S20–S22 for full models). Our predictors in each model were country (USA vs. New Zealand), internalization, symbolization, and political ideology, along with interactions between political ideology and both internalization and symbolization (due to the low rates of defiance in the New Zealand sample, we did not test for any three‐way interactions with country).

For restrictions, the model explained between 6.52% (Cox and Snell *R* ^2^) and 13.9% (Nadelkerke *R* ^2^) of the variance in responses, χ ^2^(6, *N* = 1,971) = 132.51, *p* < .001. Participants from the USA (Wald = 4.84, *p* < .001, *OR* = 3.45, 95% CI [2.14, 5.86]) and more conservative participants (Wald = −2.91, *p* = .004, *OR* = 0.68, 95% CI [0.53, 0.88]) were significantly more likely to oppose restrictions. Those higher in internalization were less likely to express opposition (Wald = −5.73, *p* < .001, *OR* = 0.93, 95% CI [0.91, 0.96]). There was a significant Internalization × Political Ideology interaction (Wald = −2.91, *p* = .004, *OR* = 0.68, 95% CI [0.53, 0.88]), such that the negative relationship between internalization and opposition was most pronounced for liberal participants (see Figure S10).

For physical contact, the model explained between 3.9% (Cox and Snell *R* ^2^) and 13.11% (Nadelkerke *R* ^2^) of the variance in responses, χ ^2^(6, *N* = 1,971) = 78.19, *p* < .001. Only country and internalization were significant predictors, with participants from the USA more likely (Wald = 2.94, *p* = .003, *OR* = 2.92, 95% CI [1.5, 6.38]) and those higher in internalization less likely (Wald = −4.32, *p* < .001, *OR* = 0.93, 95% CI [0.9, 0.96]) to oppose physical contact measures.

Finally, for hygiene adherence, the model explained between 4.91% (Cox and Snell *R* ^2^) and 13.85% (Nadelkerke *R* ^2^) of the variance in responses, χ ^2^(6, *N* = 1,971) = 98.85, *p* < .001. Country was not a significant predictor. Participants higher in internalization (Wald = −3.81, *p* < .001, *OR* = 0.95, 95% CI [0.93, 0.98]) and symbolization (Wald = −3.81, *p* < .001, *OR* = 0.95, 95% CI [0.93, 0.98]) were less likely to express opposition. Although ideology was not a significant predictor, there was a significant Internalization × Political Ideology interaction (Wald = 2.64, *p* = .008, *OR* = 1.01, 95% CI [1, 1.01]). Again, the negative relationship between internalization and opposition was most pronounced for liberal participants (see Figure S11).

## DISCUSSION

The present research tested whether moral identity predicted support for COVID‐19 mitigation measures, and whether this association was moderated by political ideology and country. The results for each hypothesis are summarized in Table 6.

**Table 6:** Summary of Support for Hypotheses for Each Measure

	Predictor	Restrictions Support	Distancing Adherence	Hygiene Adherence
H1a	Internalization	✓	✓	✓
	USA: internalization	✓	✓	✓
	NZL: internalization	✓	✓	✗
H1b	Symbolization	✓	✓	✓
	USA: symbolization	✓	✓	✓
	NZL: symbolization	✗	✗	✓
H2a	Country × Internalization × Political Ideology	✓	✓	✗
H2b	Country × Symbolization × Political Ideology	✗	✗	✗
H3a	USA: Internalization × Political Ideology	✓	✓	✓
H3b	USA: Symbolization × Political Ideology	✓	✓	✗
H4a	NZL: No Internalization × Political Ideology	✓	✓	✗
H4b	NZL: No Symbolization × Political Ideology	✓	✓	✓

*Note*: NZL, New Zealand.

The hypothesis that *internalization* would predict support/adherence (H1a) was supported, whereby internalization predicted support for all measures. We note that including country in the model changed some of the main effects, reflecting some differences between countries. For instance, in the USA sample, internalization positively predicted support for all measures, whereas in the New Zealand sample, internalization positively predicted restrictions support and distancing measures, but not hygiene measures. The hypothesis that *symbolization* would predict support/adherence (H1b) was also supported. For the total sample, symbolization positively predicted support for all measures. Again, we note some changes when country was included in the model. In the USA sample, symbolization positively predicted support for all measures, whereas in the New Zealand sample it predicted support for hygiene measures only.

Importantly, we found that the relationship between internalization and both restrictions support and distancing adherence depended on country and political ideology. However, this was not the case for hygiene adherence, partially supporting the hypothesis that the relationship between internalization and adherence is moderated by country and political ideology (H2a). For symbolization, the positive relationship between symbolization and support for mitigation measures did not interact with country and political ideology; thus, H2b is not supported.

Looking at the USA and New Zealand samples separately, in the USA, consistent with our hypotheses, the positive relationship between internalization and all three measures depended on political ideology (supporting H3a), with liberals and moderates showing greater support the higher they were in internalization, whereas this relationship was not as strong (or even reversed; see the Restrictions support section) for conservatives. For physical hygiene, greater internalization also predicted greater adherence for liberals and moderates, but conservatives showed strong adherence regardless of internalization. Further, in the USA, we observed that symbolization predicted restrictions support more for conservatives than for liberals, whereas it predicted distancing adherence more for liberals than conservatives; no such interaction was found for hygiene adherence (partially supporting H3b). In the New Zealand sample, in line with expectations, the positive relationship of symbolization and restrictions support or distancing adherence did not depend on political ideology, though this interaction was observed for hygiene adherence (greater internalization predicted greater adherence in liberals and moderates, but not in conservatives).

We conducted additional exploratory analyses examining opposition to the mitigation measures. Opposition to measures was consistently predicted by lower internalization. The negative relationship between internalization and opposition to both restrictions and physical hygiene measures was most pronounced for liberal participants. Overall, however, opposition was low, possibly reflecting that data collection took place early in the pandemic.

### Relevance for Existing Literature

These findings are consistent with previous work linking internalization with pro‐social behavior (Reed et al., 2007); we found that higher internalization was generally associated with a pro‐social tendency to support measures that were claimed to be necessary to keep the community safe, as evidenced by greater support for mitigation measures. However, we also show that this effect depends on political ideology, which is consistent with previous work on the influence of political ideology on the motivating effect of internalization (Gotowiec, 2019; Winterich et al., 2013).

Furthermore, we extend this previous work and identify national context as a key moderator of this interaction. The results show that in a context where polarization is high, such as the USA, political ideology appears to have a greater influence on the link between moral identity and support for mitigation measures than in a context where polarization is less extreme, such as New Zealand. This was evident in the observed three‐way interactions between political ideology, moral identity, and country (H2a), and further supported when looking at the results for each country separately (see H3 and H4, Tables 5 and 6). This is in line with research by Bayes et al. (2020), who identified moral values and ingroup norms (as well as accuracy motivations) as influential motivators in motivated reasoning relating to political issues. They demonstrated that the relative importance of these factors can by influenced by experimentally manipulating their relative salience (Bayes et al., 2020). We show that where polarization is high, and thus political (and associated ingroup and outgroup) identities are salient (see Iyengar & Krupenkin, 2018), internalization and political ideology interact to predict attitudes toward COVID‐19 mitigation measures.

### Internalization versus Symbolization

When developing our hypotheses, we predicted similar effects for internalization and symbolization; however, our analyses revealed differing results for the two aspects of moral identity. Across two different contexts, we found consistent but divergent relationships between political ideology and each aspect of moral identity. In both the USA and New Zealand, higher internalization was associated with a more liberal ideology, whereas higher symbolization was associated with a more conservative political ideology (see Tables 1, 2, 3).

To our knowledge, these relationships between internalization, symbolization, and political ideology have not been addressed in previous research. However, patterns of association consistent with our results have been observed. Aquino and Reed (2002) found religiosity to be positively correlated with symbolization, but unrelated to internalization. Conversely, Reed and Aquino (2003) found that internalization, but not symbolization, is positively related to perceived moral obligations toward outgroups. In two studies, Smith et al. (2014) reported significant positive associations between internalization and political liberalism. Finally, Dawson et al. (2021) observed positive relationships between internalization and the individualizing moral foundations of harm and fairness, and a similar relationship between symbolization and the binding foundations (loyalty, authority, and purity). Building upon this pattern of findings, our results are highly suggestive of a stable relationship between each aspect of moral identity and political ideology. This promises to be a fruitful direction for future study into the intersection of moral identity and political identity, and the development of political identity.

Internalization appeared to be a more consistent predictor of support for mitigation measures than symbolization, predicting support for all measures in the USA, and restrictions support and distancing adherence (but not hygiene adherence) in New Zealand. Symbolization also predicted support for all measures in the USA, but in New Zealand symbolization predicted only hygiene adherence. This may have been due to a ceiling effect in support for the other two measures in New Zealand. Additionally, the predicted three‐way interaction (with country and political ideology) in predicting support for mitigation measures was only observed for internalization, and not for symbolization.

This is consistent with accounts of the interaction between internalization and situational factors (e.g., affective polarization) put forward by Reynolds and Ceranic (2007) and Aquino et al. (2009). The former proposed that where there is a lack of social consensus for a given behavior, those high in internalization are more likely than others to act in accordance with personal, rather than social, moral values. In contrast, Aquino et al. (2009) suggested that certain situational factors can decrease the accessibility of moral identity in working memory, with a greater effect on the behavior of high internalizers for whom moral identity is normally highly accessible. Both mechanisms would lead to the outcomes observed in the present study in which a situational factor in the form of polarization contributed to different behavioral outcomes for those high in internalization, but not symbolization.

Our findings suggest that moral identity, both internalization and symbolization, can play important roles in guiding behavior. In particular, internalization was positively associated with higher levels of support for all three COVID‐19 mitigation measures, whereas symbolization only consistently predicted adherence to physical hygiene recommendations. This provides some initial evidence that framing COVID‐19 health messaging to appeal to one's moral identity, the need to act for the greater good, increases adherence to behavior that is claimed to serve that goal. However, this might be more effective for individuals who hold liberal views when polarization is higher, as observed in the USA sample.

### Differences Depending on Political Ideology and National Context

In New Zealand, internalization predicted restrictions support and distancing adherence regardless of an individual's political ideology. This suggests in this nation, support for school and hospitality closures or movement restrictions was seen as largely consistent with the moral norms of citizens. In the USA, however, these relationships were influenced by political ideology. For liberals, and individuals who did not strongly identify as either liberal or conservative (i.e., moderates), we observed a pattern similar to the New Zealand sample; internalization predicted restrictions support and distancing adherence. However, the effect was reversed for conservatives—but only in response to restrictions support. In the USA, those with a more conservative ideology who scored higher in internalization reported lower support of restrictions. In this sample, conservatives with a low internalized moral identity reported *higher* levels of restrictions support than liberals and moderates, suggesting that for these people something other than personally held morality or political ideology motivated restrictions support.

The observed interaction between political ideology and symbolization potentially provides further insight into this finding. Conservatives high in symbolization showed restrictions support similar to that of high symbolization liberals, whereas conservatives low in symbolization showed considerably less support (see Figure 2). It may be that polarization in the USA made the tension between business interests and public health salient, and prioritizing public health was perceived as being *seen by others* as the moral thing to do.

Of particular interest are the results for hygiene adherence. Internalization predicted greater reported adherence for liberals and moderates, but not for conservatives. Interestingly, though, while conservatives' adherence to physical hygiene measures appears unrelated to internalization, conservatives appear to generally show greater adherence to physical hygiene measures (we also note that physical hygiene was the only measure that found more support in the USA than in New Zealand; see Table 4). Once again, examination of the role of symbolization may provide some insight into this result. Across all political ideologies, symbolization predicted reported hygiene adherence. As with restrictions support, it may be that for all participants, and particularly conservatives, being seen abiding by social hygiene norms is a higher motivating factor than personal morality or political ideology. This may be explained by the fact that social norms around handwashing or coughing and sneezing predate the emergence of COVID‐19. It is also consistent with research linking conservatives' concern for purity and cleanliness (Graham et al., 2012).

### Limitations and Future Directions

The results are complicated by the fluid nature of COVID‐19 mitigation procedures that also differ between nations. For example, at the time of this writing, New Zealand had entered strict lockdowns due to rising outbreaks of the Delta variant—it is unclear whether sustained lockdowns will impact the adherence trends reported in this article. Of course, this research was conducted at the beginning of the pandemic wherein populations and governments lacked knowledge on the virus; the resulting behaviors may therefore not reflect population adherence to mitigation procedures later in time.

Although our investigation focused on the potential role of affective polarization in behavioral responses to COVID‐19 mitigation measures, we do not rule out the possibility that other differences between the USA and New Zealand may contribute to these outcomes. In addition to the differences in their electoral systems outlined earlier, the two countries differ vastly on other characteristics, including population (New Zealand = 5.1 million, USA = 329.5 million in 2020) and GDP per capita (New Zealand = USD41,791.79, USA = USD63,543.58 in 2020). New Zealand also has a history of comparatively early adoption of “liberal” social change, including being the first self‐governing country to offer full suffrage to women and universally franchising their First Nation (Mãori) men as early as 1867. While we are unaware of any research linking these country‐level factors to individual COVID‐19 behavioral responses, we recommend them as a direction for future study in order to get a more complete picture of transnational COVID behavior.

Given that in both countries in this study, internalization was a relatively consistent predictor of support for all mitigation measures, future research should endeavor to disentangle the likely mixed effects of moral symbolization versus internalization across more representative samples, ideally with real‐time data collection built into study designs. Our study is a cross‐sectional correlational design, limiting our ability to make inferences regarding directionality (i.e., whether moral identity influences political ideology or vice versa). Longitudinal or experimental data are needed to test the direction of relationships between these variables. We note also that our measures were self‐reported, and this may not mirror people's actual behaviors.

We measured political ideology using a single item that assumes a unidimensional construct. However, growing evidence suggests a multidimensional conceptualization of political ideology is more appropriate (e.g., Treier & Hillygus, 2009). Future research should test how the various dimensions of political ideology may differentially interact with moral identity in guiding behavior.

We note that the effects reported here are comparably small, potentially limiting the practical applicability of this work. It is also worth noting that the New Zealand sample was comparatively smaller than the USA sample (*n* = 509 versus *n* = 1,471), which may have resulted in lower power to detect significant effects should they exist in the New Zealand sample, in particular in relation to detecting a significant interaction between moral identity and political ideology on adherence (H4a and H4b). Further research is needed to replicate this finding with a larger sample. Despite this, our findings further our understanding of how moral identity and political ideology interact to shape attitudes and behavioral intentions. These insights can inform future related work, highlighting important considerations when addressing issues that may become moralized. Furthermore, we note that the effects of interest are of comparable size to other variables that have emerged as relevant in the wider literature (see full tables in the online appendix for comparisons), such as national identification (Van Bavel et al., 2022) or conspiracy beliefs (Earnshaw et al., 2020).

## CONCLUSION

Using secondary data from a large‐scale international collaboration, we tested specific hypotheses regarding the roles of moral identity, political ideology, and national context in predicting people's reported adherence to and support for distinct COVID‐19 mitigation measures. Our hypotheses received mixed support. The findings highlight the significant challenges associated with establishing new norms in an uncertain, complex, and evolving situation such as that posed by the COVID‐19 pandemic. We present initial evidence that in contexts where polarization is less extreme, there may be value in appealing to people's internal moral compass; however, this becomes more complicated in nations experiencing greater levels of affective polarization. This means that, counter to intuitive wisdom, framing COVID‐19 as a moral issue does not guarantee success and may even undermine compliance with the mitigation COVID‐19 measures. Moral framing may lead people to draw parallels to their own moral values (e.g., fairness/equality versus individual freedoms; see Feinberg & Willer, 2019), reinforcing existing disagreements.

## Supporting information


**Figure S1**. Residuals by country, predicting restrictions support.
**Figure S2**. Residuals by country, predicting adherence to physical distancing measures.
**Figure S3**. Residuals by country, predicting adherence to physical hygiene measures.
**Table S1**. Participant Characteristics (*N* = 1,980)
**Table S2**. Correlations Between All Variables Including Covariates for the Total Sample
**Table S3**. Correlations Between All Variables Including Covariates for the USA Subsample
**Table S4**. Correlations Between All Variables Including Covariates for the New Zealand Subsample
**Table S5**. Means, Standard Deviations, and Differences Between the Subsamples
**Table S6**. Means, Standard Deviations, and Differences Depending on Gender
**Table S7**. Model 1: Full Regression Model Predicting Restrictions Support (Without Country
**Table S8**. Model 2: Full Regression Model Predicting Restrictions Support (With Country Included as a Variable)
**Figure S4**. The relationship between political ideology and internalization in predicting restrictions support for the total sample.
**Figure S5**. The relationship between political ideology and symbolization in predicting restrictions support for the total sample.
**Table S9**. Model 3: Predictors of Restrictions Support in USA Subsample
**Table S10**. Model 4: Predictors of Restrictions Support in New Zealand Subsample
**Table S11**. Model 5: Full Regression Model Predicting Adherence to Physical Distancing Measures (Without Country)
**Table S12**. Model 6: Full Model Predicting Adherence to Physical Distancing Measures (With Country Included as a Variable)
**Figure S6**. The relationship between political ideology and internalization in predicting physical distancing for the total sample.
**Figure S7**. The relationship between political ideology and symbolization in predicting physical distancing for the total sample.
**Table S13**. Model 7: Predictors of Adherence to Physical Distancing Measures in the USA Subsample
**Table S14**. Model 8: Predictors of Adherence to Physical Distancing Measures in the New Zealand Subsample
**Table S15**. Model 9: Full Regression Model Predicting Adherence to Physical Hygiene Measures (Wwithout Country)
**Table S16**. Model 10: Full Model Predicting Adherence to Physical Hygiene Measures (With Country Included as a Variable)
**Figure S8**. The relationship between political ideology and internalization predicting physical hygiene for the total sample.
**Figure S9**. The relationship between political ideology and moral identity symbolization in predicting physical hygiene for the total sample.
**Table S17**. Model 11: Predictors of Adherence to Physical Hygiene Measures in the USA Subsample
**Table S18**. Model 12: Predictors of Adherence to Physical Hygiene Measures in the New Zealand Subsample
**Table S19**. Summary of Support for Hypotheses for Each Measure
**Table S20**. Predictors of Opposition to Restrictions
**Figure S10**. Interaction between political ideology and moral identity internalization in predicting opposition to restrictions.
**Table S21**. Predictors of Opposition to Physical Contact Measures
**Table S22**. Predictors of Opposition to Physical Hygiene Measures
**Figure S11**. Interaction between political ideology and moral identity internalization in predicting opposition to hygiene measures.

## Footnotes

1 We also note that ideological differences in support for COVID‐19 mitigation measures may not necessarily depend on moral values. That is, liberals may simply be more likely to support the measures because they are more supportive of government intervention more generally (Campbell & Kay, 2014). This being the case, we would not expect moral identity to influence support for mitigation measures or to interact with political ideology in influencing support for the same.

^2^
McHugh, Griffin, and McCashin participated in the ICSMP project making up the Irish team; for more information, see https://icsmp‐covid19.netlify.app/index.html.
