# Supplementary material for: Moral Identity Predicts Adherence to COVID‐19 Mitigation Procedures Depending on Political Ideology: A Comparison Between the USA and New Zealand
Source: Polit Psychol. 2022 Jun 17:10.1111/pops.12838. Online ahead of print. doi: 10.1111/pops.12838 (PMC9349772; doi:10.1111/pops.12838)

Online Appendix

Supplementary Materials

Restriction Support Items

During the days of the coronavirus (COVID-19) pandemic, I have been ...

- In favor of closing all schools and universities
- In favor of closing all bars and restaurants
- In favor of closing all parks
- In favor of forbidding all public gatherings where many people are gathered at one place (sports and culture)
- In favor of forbidding all non-necessary travel

Distancing Adherence Items

During the days of the coronavirus (COVID-19) pandemic, I have been ...

- Staying at home as much as practically possible
- Visiting friends, family, or colleagues outside my home
- Keeping the number of grocery store visits at an absolute minimum
- Keeping physical distance from all other people outside my home
- Avoiding handshaking with people outside my home

Hygiene Adherence Items

During the days of the coronavirus (COVID-19) pandemic, I have been ...

- Washing my hands longer than usual
- Washing my hands (with soap) more thoroughly than usual
- Always washing my hands immediately after returning home
- Disinfecting frequently used objects, such as mobile phones and keys

- Sneezing and coughing into my upper sleeve

Moral Identity Items

The items measuring moral identity are listed below. Items are denoted in parenthesis as "I" (internalization) and "S" (symbolization). Reverse coded items are labelled with "(R)".

"*Listed below are some characteristics that might describe a person: caring, compassionate, fair, friendly, generous, helpful, hardworking, honest, kind. The person with these characteristics could be you or it could be someone else. For a moment, visualize in your mind the kind of person who has these characteristics. Imagine how that person would think, feel, and act. When you have a clear image of what this person would be like, answer the following questions.*"

(I) It would make me feel good to be a person who has these characteristics.

(I) Being someone who has these characteristics is an important part of who I am.

(S) I often wear clothes that identify me as having these characteristics.

(I) I would be ashamed to be a person who had these characteristics. (R)

(S) The types of things I do in my spare time (e.g., hobbies) clearly identify me as having these characteristics

(S) The kinds of books and magazines that I read identify me as having these characteristics.

(I) Having these characteristics is not really important to me. (R)

(S) The fact that I have these characteristics is communicated to others by my membership in certain organizations.

(S) I am actively involved in activities that communicate to others that I have these characteristics.

(I) I strongly desire to have these characteristics.

Moral Circle

On this page, we would like you to indicate the extent of your moral circle. By moral circle, we mean the circle of people or other entities for which you are concerned about right and wrong done toward them. Please use the following scale to select the extent of your moral circle:

1 - all of your immediate family

2 - all of your extended family

3 - all of your closest friends

4 - all of your friends (including distant ones)

5 - all of your acquaintances

6 - all people you have ever met

7 - all people in your country

8 - all people on your continent

9 - all people on all continents

10 - all mammals

11 - all amphibians, reptiles, mammals, fish, and birds

12 - all animals on earth, including paramecia and amoebae

13 - all animals in the universe, including alien lifeforms

14 - all living things in the universe, including plants and trees

15 - all natural things in the universe, including inert entities such as rocks

16 - all things in existence

Please select the number that represents the extent of your moral circle. Note that, in this scale, the number you select includes all the numbers below it as well. So, for example, if you select 10 (all mammals) you are also including number 1-9 (up to "all people on all continents") in your moral circle.

Supplementary Results

Initial testing revealed that for restrictions support, allowing intercepts to vary with country significantly improved the predictive power of the model, $\chi 2(1) = 8290.99$, $p < .001$.

Similarly, for distancing adherence, allowing intercepts to vary with country significantly improved the predictive power of the model, $\chi^2(1) = 3951.04$, $p < .001$.

Finally, for hygiene adherence, allowing intercepts to vary with country significantly improved the predictive power of the model, $\chi^2(1) = 4677.74$, $p < .001$. The country level residuals for the full models for each dependent variable are plotted below.

Residual Plots

Figure S1

Residuals by country, predicting restrictions support

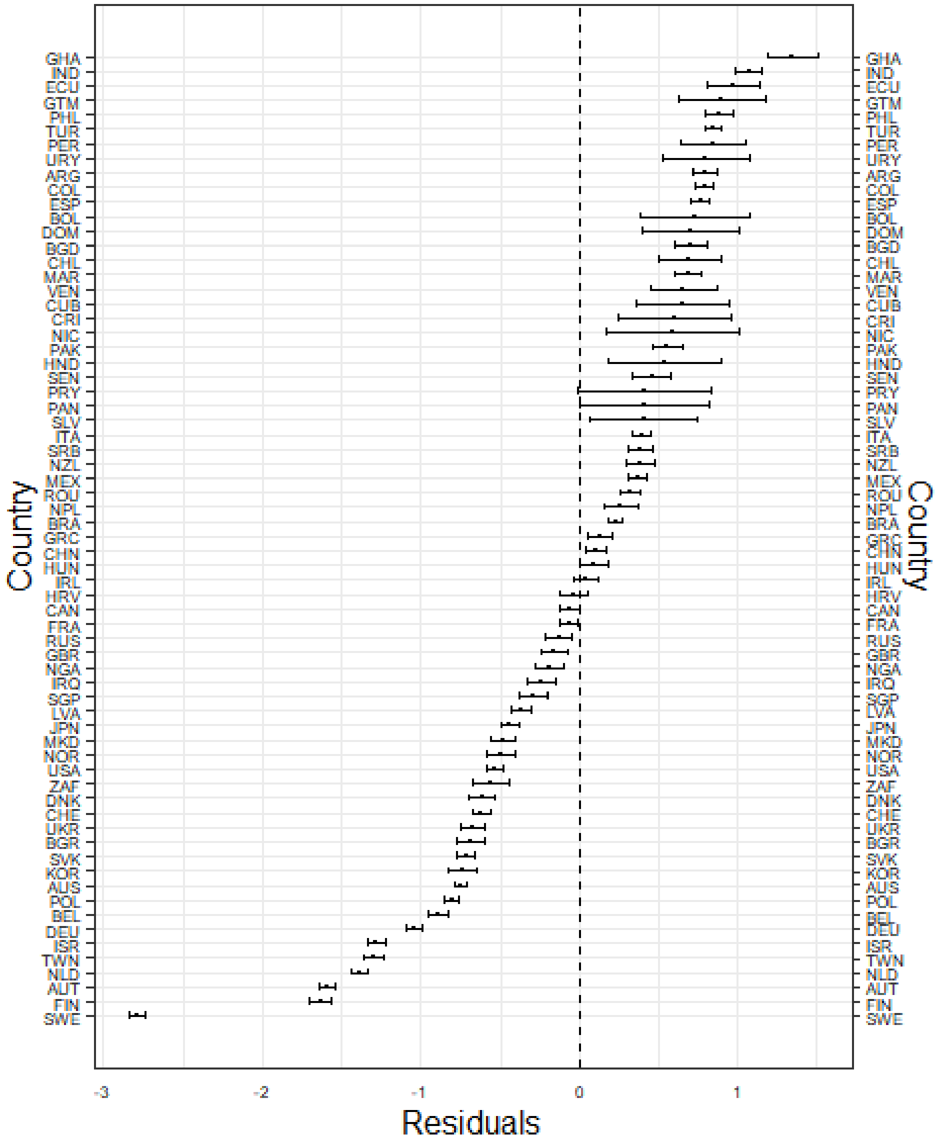

Figure S2

Residuals by country, predicting adherence to physical distancing measures

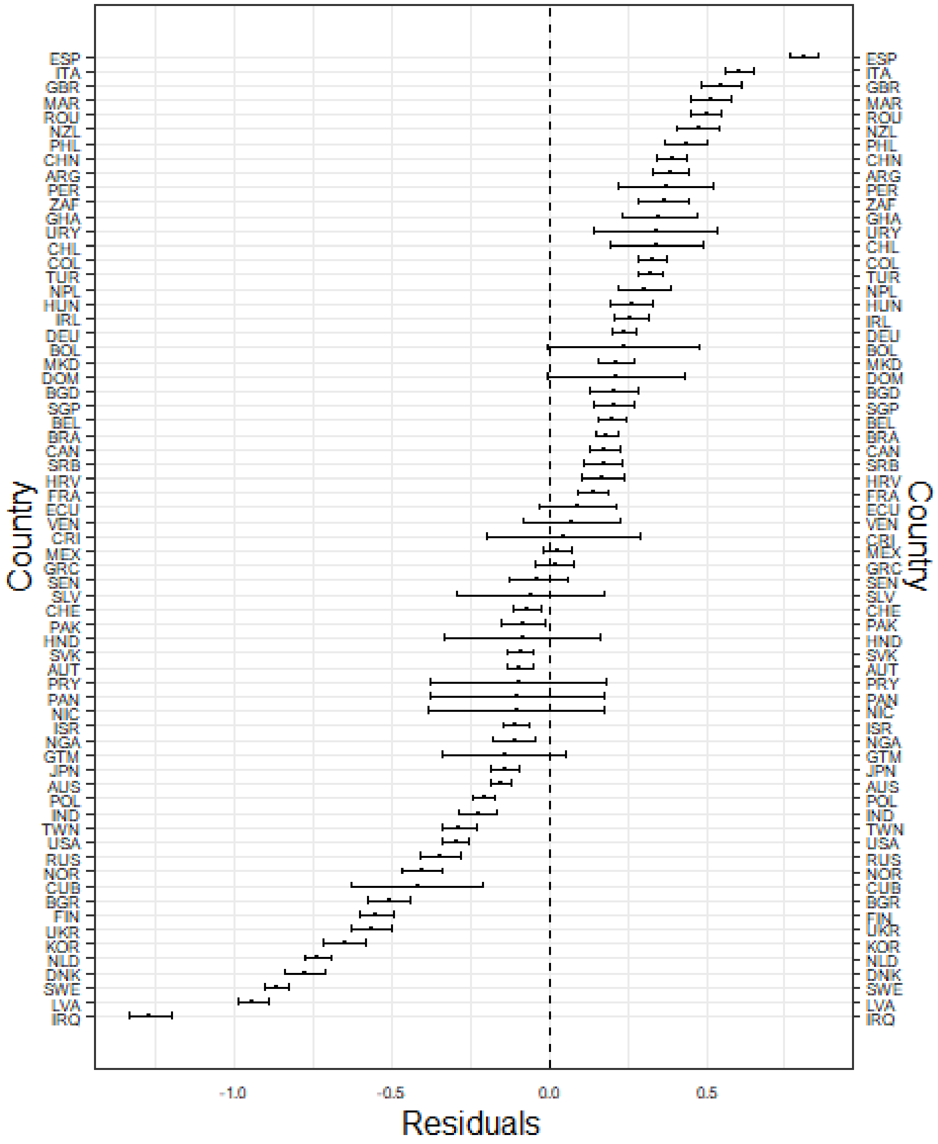

Figure S3

Residuals by country, predicting adherence to physical hygiene measures

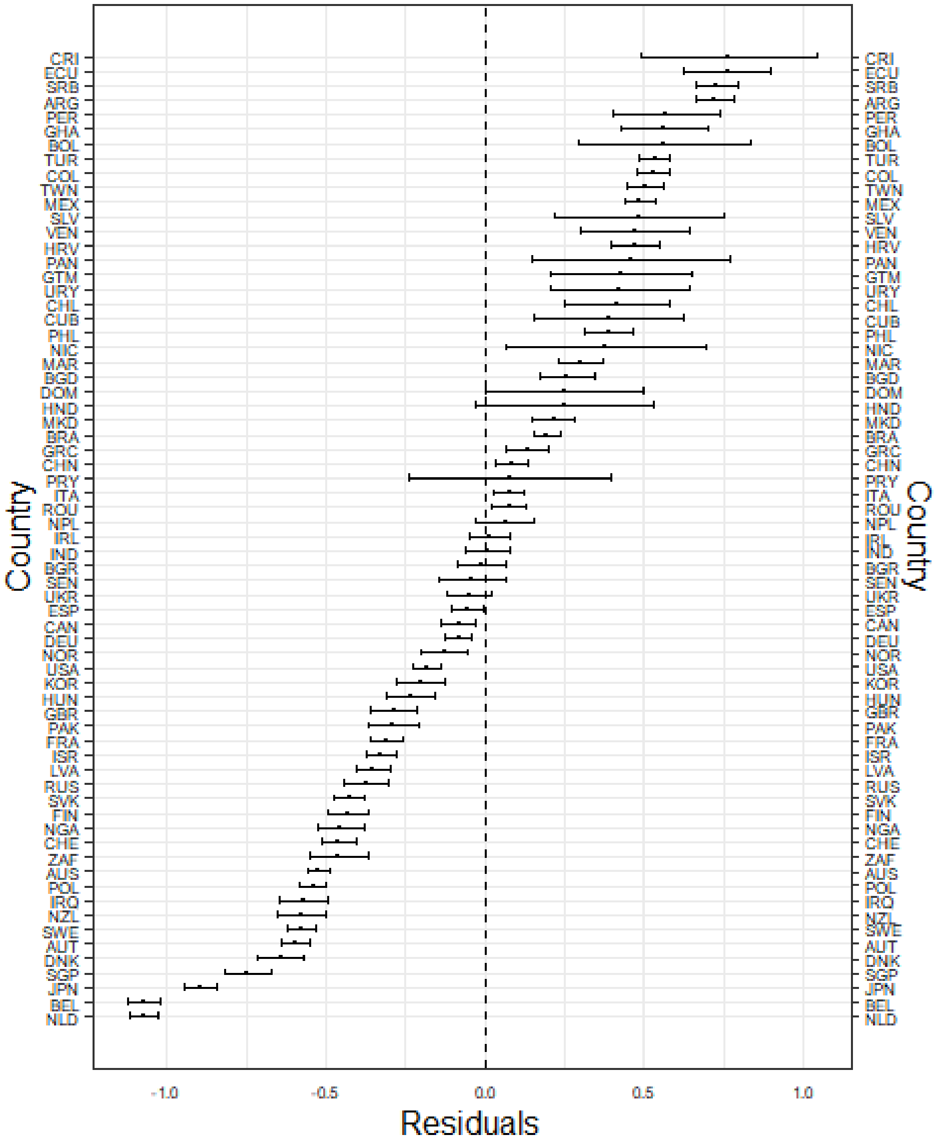

Participant Characteristics

Table S1

Participant Characteristics (N = 1980)

	USA (*N* = 1471)			New Zealand (*N* = 509)		
	M (range)	*SD*	*N (%)*	*M (range)*	*SD*	*N (%)*
Male			755 (51.3%)			253 (49.7%)
Female			710 (48.3%)			255 (50.1%)
Other gender (missing)			4(2)			1
Age	44.32 (18-86)	16.43		45.78 (18-89)	17.63	

Correlations, Group Means, and Standard Deviations

Table S2

Correlations between all variables including covariates for the total sample

	**1**	**2**	**3**	**4**	**5**	**6**	**7**	**8**	**9**	**10**	**11**	**12**	**13**	***M***	***SD***
1. Restrictions Support	-													7.82	2.30
2. Distancing Adherence	.55**	-												8.13	1.77
3. Hygiene Adherence	.41**	.39**	-											7.86	1.90
4. Age	.15**	.30**	.08**	-										44.70	16.76
5. Risk Perception	.09**	-.13**	.12**	-.20**	-									44.96	26.74
6. Narcissism Admiration	.04	-.21**	.17**	-.33**	.25**	-								5.20	2.55
7. Narcissism Rivalry	-.06*	-.38**	-.04*	-.34**	.37**	.59**	-							3.80	2.64
8. Collective Narcissism	.07*	-.12**	.23**	-.08**	.16**	.49**	.40**	-						5.53	2.57
9. Conspiracy Beliefs	-.20**	-.38**	.01	-.30**	.29**	.47**	.53**	.47**	-					3.81	3.10
10. National Identification	.23**	.26**	.32**	.24**	.00	.12**	-.05*	.35**	.05*	-				8.14	2.14
11. Moral Circle	.15**	.16**	.10**	.10**	.01	-.05*	-.10**	-.07*	-.12**	.04	-			8.92	5.21
12. Internalization	.30**	.53**	.27**	.30**	-.22**	-.24**	-.52**	-.20**	-.41**	.27**	.22**	-		7.44	1.86
13. Symbolization	.16**	-.03	.33**	-.17**	.21**	.52**	.32**	.41**	.36**	.21**	.04*	-.02	-	5.89	2.10
14. Political Ideology	-.10**	-.15**	.12**	-.10**	.16**	.34**	.32**	.46**	.44**	.23**	-.10**	-.16**	.31**	5.89	2.58

Note: * = sig. at p < .05; ** = sig. at p < .001

Table S3

Correlations between all variables including covariates for the USA subsample

	**1**	**2**	**3**	**4**	**5**	**6**	**7**	**8**	**9**	**10**	**11**	**12**	**13**	***M***	***SD***
1. Restrictions Support	-													7.58	2.39
2. Distancing Adherence	.54**	-												7.89	1.81
3. Hygiene Adherence	.48**	.50**	-											7.99	1.90
4. Age	.15**	.32**	.12**	-										44.32	16.43
5. Risk Perception	.19**	-.04	.13**	-.19**	-									49.17	26.47
6. Narcissism Admiration	.11**	-.17**	.17**	-.35**	.24**	-								5.42	2.60
7. Narcissism Rivalry	-.02	-.36**	-.05*	-.36**	.40**	.60**	-							3.94	2.76
8. Collective Narcissism	.08*	-.13**	.23**	-.07*	.17**	.52**	.44**	-						5.54	2.68
9. Conspiracy Beliefs	-.17**	-.37**	-.02	-.32**	.25**	.49**	.56**	.51**	-					4.09	3.13
10. National Identification	.23**	.29**	.37**	.26**	.00	.14**	-.03	.40**	.07*	-				8.13	2.13
11. Moral Circle	.17**	.19**	.10**	.14**	.00	-.05*	-.11**	-.10**	-.14**	.04	-			8.94	5.19
12. Internalization	.28**	.55**	.31**	.34**	-.22**	-.24**	-.53**	-.22**	-.44**	.29**	.21**	-		7.37	1.91
13. Symbolization	.23**	.03	.33**	-.20**	.22**	.54**	.35**	.43**	.35**	.22**	.04	-.02	-	6.07	2.13
14. Political Ideology	-.07*	-.12**	.14**	-.09**	.12**	.35**	.32**	.54**	.46**	.30**	-.10**	-.14**	.33**	6.11	2.69

Note: * = sig. at $p < .05$; ** = sig. at $p < .001$

Table S4

Correlations between all variables including covariates for the New Zealand subsample

	**1**	**2**	**3**	**4**	**5**	**6**	**7**	**8**	**9**	**10**	**11**	**12**	**13**	***M***	***SD***
1. Restrictions Support	-													8.53	1.84
2. Distancing Adherence	.50**	-												8.84	1.45
3. Hygiene Adherence	.28**	.20**	-											7.50	1.85
4. Age	.12*	.26**	-.02	-										45.78	17.63
5. Risk Perception	-.05	-.18**	.00	-.21**	-									32.80	23.60
6. Narcissism Admiration	-.08	-.24**	.12*	-.28**	.12*	-								4.56	2.29
7. Narcissism Rivalry	-.16**	-.41**	-.07	-.27**	.21**	.56**	-							3.38	2.22
8. Collective Narcissism	.07	-.09*	.22**	-.13*	.14*	.40**	.25**	-						5.48	2.24
9. Conspiracy Beliefs	-.24**	-.31**	.06	-.24**	.31**	.33**	.35**	.36**	-					2.99	2.85
10. National Identification	.23**	.21**	.19**	.17**	-.02	.06	-.10*	.20**	-.01	-				8.17	2.17
11. Moral Circle	.11*	.08	.09	.00	.01	-.06	-.07	.02	-.07	.06	-			8.85	5.27
12. Internalization	.33**	.45**	.18**	.18**	-.17**	-.21**	-.47**	-.12*	-.26**	.23**	.24**	-		7.64	1.70
13. Symbolization	.05	-.10*	.30**	-.09*	.05	.40**	.16**	.37**	.33**	.20**	.05	.04	-	5.37	1.91
14. Political Ideology	-.10*	-.11*	-.01	-.10*	.14*	.25**	.27**	.14*	.27**	.02	-.11*	-.22**	.14*	5.23	2.12

Note: * = sig. at $p < .05$; ** = sig. at $p < .001$

Table S5

Means Standard Deviations and Differences between the Subsamples

	USA		NZL					
	M	*SD*	*M*	*SD*	*t*	*df*	*p*	*d*
Restrictions Support	7.58	2.39	8.53	1.84	9.22	1138.13	< .001**	0.42
Distancing Adherence	7.89	1.81	8.84	1.45	11.85	1092.8	< .001**	0.55
Hygiene Adherence	7.99	1.9	7.5	1.85	-5.11	903.28	< .001**	0.26
Age	44.32	16.43	45.78	17.63	1.63	834.07	.103	0.09
Risk Perception	49.17	26.47	32.8	23.6	-13.05	980.39	< .001**	0.64
Narcissism Admiration	5.42	2.6	4.56	2.29	-7.04	992.51	< .001**	0.34
Narcissism Rivalry	3.94	2.76	3.38	2.22	-4.62	1085.6	< .001**	0.21
Conspiracy Beliefs	4.09	3.13	2.99	2.85	-7.27	961.03	< .001**	0.36
National Identification	8.13	2.13	8.17	2.17	0.37	866.61	.709	0.02
Moral Circle	8.94	5.19	8.85	5.27	-0.35	871.89	.727	0.02
Moral Identity Internalization	7.37	1.91	7.64	1.7	3.02	982.03	.003*	0.15
Moral Identity Symbolization	6.07	2.13	5.37	1.91	-6.84	980.43	< .001**	0.33
Political Ideology	6.11	2.69	5.23	2.12	-7.53	1105.91	< .001**	0.35

Note: NZL = New Zealand; * = sig. at $p < .05$; ** = sig. at $p < .001$

Table S6

Means Standard Deviations and Differences depending on Gender

	Female		Male		Other					
	M	*SD*	*M*	*SD*	*M*	*SD*	*F*	*df*	*p*	η_p^2
Restrictions Support	7.67	2.36	7.97	2.23	7.64	1.73	4.18	2, 1975	.015*	.004
Distancing Adherence	7.88	1.76	8.38	1.75	8.12	2.29	19.68	2, 1974	< .001**	.020
Hygiene Adherence	7.68	1.95	8.04	1.82	6.08	3.00	11.39	2, 1974	< .001**	.011
Age	45.53	16.42	43.96	17.04	33.2	14.77	3.33	2, 1972	.036*	.003
Risk Perception	44.24	28.52	45.67	24.89	48	31.74	0.74	2, 1972	.477	< .001
Narcissism Admiration	5.64	2.57	4.78	2.45	4.13	3.13	29.59	2, 1973	< .001**	.029
Narcissism Rivalry	4.35	2.71	3.27	2.47	3.67	2.39	43.38	2, 1972	< .001**	.042
Conspiracy Beliefs	4.15	3.23	3.48	2.93	3.00	2.90	11.56	2, 1972	< .001**	.012
National Identification	8.20	2.11	8.10	2.17	7.10	2.53	1.09	2, 1973	.337	.001
Moral Circle	8.36	5.09	9.47	5.27	6.00	3.74	11.97	2, 1970	< .001**	.012
Moral ID Internalization	7.11	1.78	7.75	1.87	7.24	2.21	30.27	2, 1967	< .001**	.030
Moral ID Symbolization	6.07	2.19	5.72	1.99	4.28	1.29	8.32	2, 1972	< .001**	.008
Political Ideology	6.24	2.63	5.55	2.48	5.6	3.51	17.74	2, 1966	< .001**	.018

Note: * = sig. at $p < .05$; ** = sig. at $p < .001$

Full Regression Tables

Restrictions Support

Table S7

Model 1: Full regression model predicting Restrictions Support (without Country); R^2 = 0.25, *S.E.* = 0.87, adjusted R^2 = 0.24, $F(17, 1932) = 37.28$, $p < .001$.

	b	*S.E.*	95% CI	$t(1932)$	p
Intercept	-0.27	0.07	[−0.41, −0.14]	-3.89	< .001
Age	0.00	0.00	[0.00, 0.01]	2.92	.004
Sex - Male/Female	0.10	0.04	[0.01, 0.18]	2.28	.022
Sex - Other/Female	0.24	0.39	[−0.53, 1.01]	0.61	.542
Risk Perception	0.12	0.02	[0.08, 0.16]	5.52	< .001
Narcissism Admiration	0.05	0.03	[−0.01, 0.10]	1.58	.114
Narcissism Rivalry	0.06	0.03	[0.00, 0.12]	2.03	.043
Collective Narcissism	0.14	0.03	[0.08, 0.19]	5.05	< .001
Conspiracy Beliefs	-0.26	0.03	[−0.32, −0.21]	-9.87	< .001
National Identification	0.10	0.02	[0.05, 0.14]	4.19	< .001
Moral Circle	0.05	0.02	[0.01, 0.09]	2.53	.012
Moral Identity Internalization	0.22	0.03	[0.16, 0.27]	8.29	< .001
Moral Identity Symbolization	0.14	0.02	[0.10, 0.19]	5.88	< .001
Political Ideology	-0.12	0.02	[−0.17, −0.08]	-5.20	< .001
Moral Circle × Internalization	0.00	0.02	[−0.05, 0.04]	-0.22	.824
Moral Circle × Symbolization	0.03	0.02	[0.00, 0.07]	1.74	.081
Internalization× Political Ideology	-0.12	0.02	[−0.16, −0.08]	-5.80	< .001
Symbolization × Political Ideology	0.10	0.02	[0.06, 0.13]	5.33	< .001

Note: Internalization = Moral Identity Internalization; Symbolization = Moral Identity Symbolization

Table S8

Model 2: Full regression model predicting Restrictions Support (with Country included a variable); R^2 = 0.30, *S.E.* = 0.84, adjusted R^2 = 0.29, $F(35, 1914) = 23.67$, $p < .001$.

	b	***S.E.***	**95% CI**	$t(1914)$	***p***
Intercept	0.05	0.12	[−0.18, 0.29]	0.45	.654
Country	-0.53	0.15	[−0.82, −0.24]	-3.59	< .001
Age	0.00	0.00	[0.00, 0.01]	0.77	.443
Sex - Male/Female	0.23	0.08	[0.07, 0.39]	2.81	.005
Sex - Other/Female	0.41	0.86	[−1.28, 2.10]	0.48	.634
Risk Perception	0.02	0.05	[−0.07, 0.12]	0.52	.601
Narcissism Admiration	-0.03	0.06	[−0.15, 0.08]	-0.60	.549
Narcissism Rivalry	0.07	0.06	[−0.05, 0.20]	1.13	.257
Collective Narcissism	0.13	0.05	[0.03, 0.23]	2.47	.013
Conspiracy Beliefs	-0.22	0.05	[−0.32, −0.13]	-4.49	< .001
National Identification	0.10	0.04	[0.02, 0.18]	2.39	.017
Moral Circle	0.00	0.04	[−0.08, 0.08]	0.09	.929
Moral Identity Internalization	0.21	0.05	[0.10, 0.31]	3.97	< .001
Moral Identity Symbolization	0.06	0.05	[−0.04, 0.15]	1.10	.270
Political Ideology	-0.02	0.05	[−0.12, 0.09]	-0.29	.769
Moral Circle × Internalization	-0.01	0.04	[−0.10, 0.07]	-0.29	.772
Moral Circle × Symbolization	0.00	0.04	[−0.08, 0.08]	0.08	.935
Internalization × Political Ideology	0.03	0.05	[−0.07, 0.13]	0.51	.608
Symbolization × Political Ideology	0.05	0.05	[−0.04, 0.14]	1.02	.306
Country × Age	0.00	0.00	[0.00, 0.01]	1.38	.169
Country × Sex - Male/Female	-0.15	0.09	[−0.33, 0.04]	-1.59	.113
Country × Sex - Other/Female	-0.04	0.96	[−1.93, 1.84]	-0.05	.963
Country × Risk perception	0.18	0.05	[0.08, 0.28]	3.39	.001
Country × Narcissism admiration	0.13	0.07	[0.00, 0.26]	1.96	.051
Country × Narcissism rivalry	-0.05	0.07	[−0.19, 0.09]	-0.64	.521
Country × Collective narcissism	-0.03	0.06	[−0.14, 0.09]	-0.43	.665
Country × Conspiracy	-0.01	0.06	[−0.12, 0.11]	-0.13	.899
Country × National Identification	0.00	0.05	[−0.10, 0.10]	0.00	.996
Country × Moral Circle	0.06	0.05	[−0.03, 0.15]	1.36	.174
Country × Internalization	0.02	0.06	[−0.10, 0.13]	0.27	.791
Country × Symbolization	0.13	0.06	[0.02, 0.24]	2.26	.024
Country × Political Ideology	-0.12	0.06	[−0.23, −0.01]	-2.06	.039
Country × M. Circle × Internalization	0.02	0.05	[−0.08, 0.11]	0.33	.739
Country × M. Circle × Symbolization	0.03	0.05	[−0.06, 0.12]	0.57	.566
Country × Internalization × P. Ideology	-0.15	0.06	[−0.26, −0.04]	-2.69	.007
Country × Symbolization × P. Ideology	0.06	0.05	[−0.03, 0.16]	1.28	.202

Note: Internalization = Moral Identity Internalization; Symbolization = Moral Identity Symbolization; M. Circle = Moral Circle; P. Ideology = Political Ideology

Figure S4

The relationship between political ideology and internalization in predicting restrictions support for the total sample.

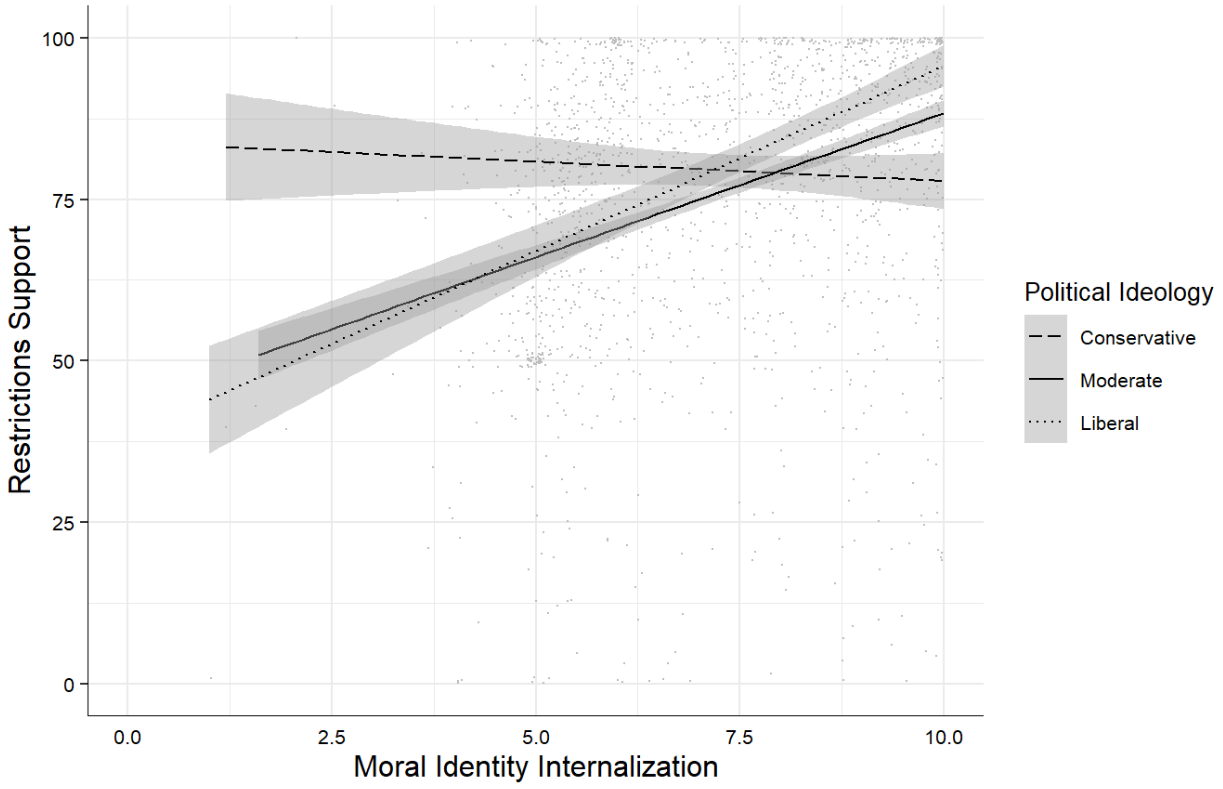

Figure S5

The relationship between political ideology and symbolization in predicting restrictions support for the total sample.

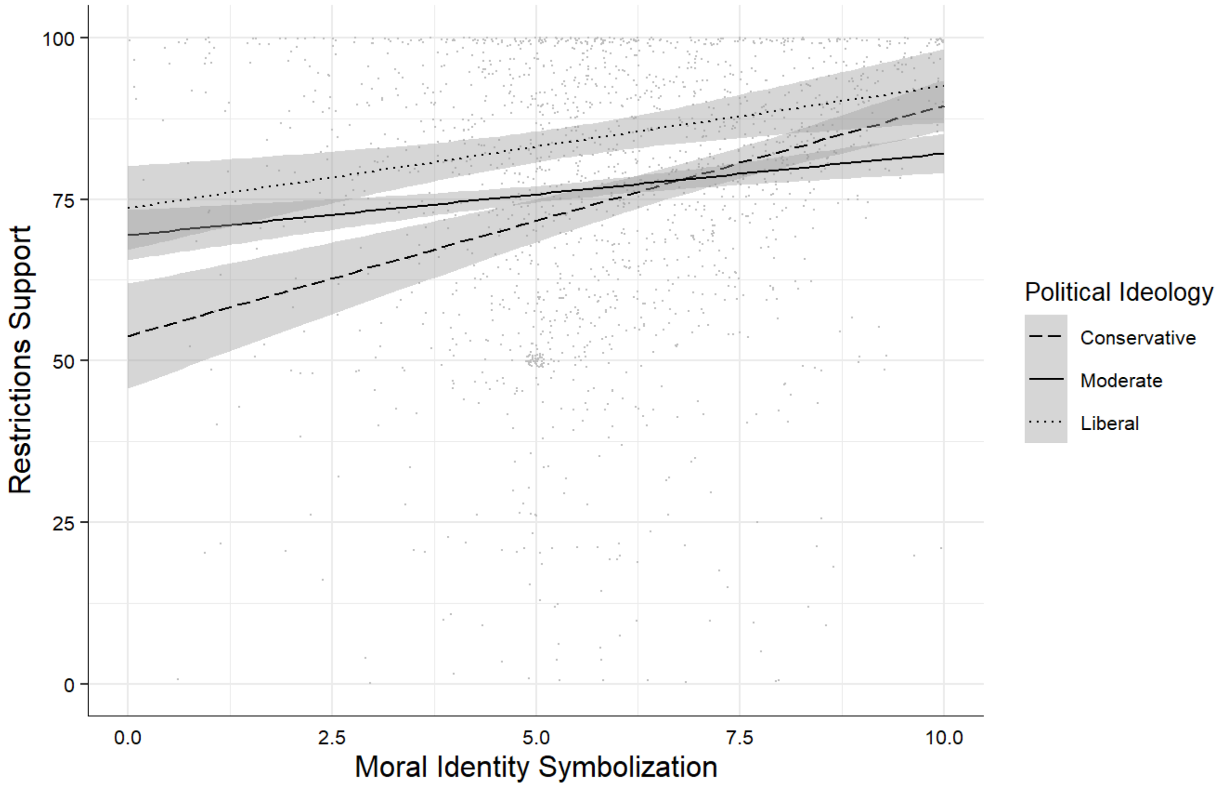

Table S9

Model 3: Predictors of Restrictions Support in USA subsample; R^2 = 0.31, *S.E.* = 0.83, adjusted R^2 = 0.3, $F(17, 1429) = 37.46$, $p < .001$.

	b	***S.E.***	**95% CI**	$t(1430)$	***p***
Intercept	-0.48	0.09	[−0.65, −0.31]	-5.46	< .001
Age	0.01	0.00	[0.00, 0.01]	3.40	.001
Sex - Male/Female	0.08	0.05	[−0.02, 0.18]	1.63	.104
Sex - Other/Female	0.37	0.44	[−0.50, 1.23]	0.83	.408
Risk Perception	0.20	0.03	[0.15, 0.26]	7.80	< .001
Narcissism Admiration	0.10	0.03	[0.03, 0.16]	2.84	.005
Narcissism Rivalry	0.03	0.03	[−0.04, 0.09]	0.76	.449
Collective Narcissism	0.10	0.03	[0.04, 0.17]	3.08	.002
Conspiracy Beliefs	-0.23	0.03	[−0.30, −0.17]	-7.21	< .001
National Identification	0.10	0.03	[0.04, 0.15]	3.33	.001
Moral Circle	0.07	0.02	[0.02, 0.11]	2.75	.006
Moral Identity Internalization	0.22	0.03	[0.16, 0.28]	7.26	< .001
Moral Identity Symbolization	0.18	0.03	[0.13, 0.24]	6.37	< .001
Political Ideology	-0.14	0.03	[−0.19, −0.08]	-4.83	< .001
Moral Circle × Internalization	0.00	0.02	[−0.04, 0.05]	0.16	.875
Moral Circle × Symbolization	0.03	0.02	[−0.01, 0.07]	1.37	.171
Internalization× Political Ideology	-0.12	0.02	[−0.17, −0.08]	-5.46	< .001
Symbolization × Political Ideology	0.11	0.02	[0.07, 0.15]	5.47	< .001

Note: Internalization = Moral Identity Internalization; Symbolization = Moral Identity Symbolization

Table S10

Model 4: Predictors of Restrictions Support in New Zealand subsample; R^2 = 0.21, *S.E.* = 0.72, adjusted R^2 = 0.18, $F(17, 484) = 7.52$, $p < .001$.

	b	***S.E.***	**95% CI**	$t(484)$	***p***
Intercept	0.05	0.10	[−0.15, 0.26]	0.52	.602
Age	0.00	0.00	[0.00, 0.01]	0.90	.371
Sex - Male/Female	0.23	0.07	[0.09, 0.36]	3.28	.001
Sex - Other/Female	0.41	0.74	[−1.04, 1.86]	0.56	.579
Risk Perception	0.02	0.04	[−0.05, 0.10]	0.61	.542
Narcissism Admiration	-0.03	0.05	[−0.13, 0.06]	-0.70	.485
Narcissism Rivalry	0.07	0.05	[−0.04, 0.18]	1.32	.187
Collective Narcissism	0.13	0.04	[0.04, 0.21]	2.89	.004
Conspiracy Beliefs	-0.22	0.04	[−0.31, −0.14]	-5.23	< .001
National Identification	0.10	0.03	[0.03, 0.17]	2.78	.006
Moral Circle	0.00	0.03	[−0.06, 0.07]	0.10	.917
Moral Identity Internalization	0.21	0.04	[0.12, 0.29]	4.63	< .001
Moral Identity Symbolization	0.06	0.04	[−0.03, 0.14]	1.29	.199
Political Ideology	-0.02	0.04	[−0.10, 0.07]	-0.34	.732
Moral Circle × Internalization	-0.01	0.04	[−0.08, 0.06]	-0.34	.735
Moral Circle × Symbolization	0.00	0.04	[−0.07, 0.07]	0.10	.924
Internalization × Political Ideology	0.03	0.04	[−0.06, 0.11]	0.60	.550
Symbolization × Political Ideology	0.05	0.04	[−0.03, 0.13]	1.19	.233

Note: Internalization = Moral Identity Internalization; Symbolization = Moral Identity Symbolization

Distancing Adherence

Table S11

Model 5: Full regression model predicting adherence to Physical Distancing measures (without Country); R^2 = 0.36, *S.E.* = 0.8, adjusted R^2 = 0.36, $F(17, 1932) = 64.68$, $p < .001$.

	b	*S.E.*	95% CI	$t(1932)$	p
Intercept	-0.34	0.06	$[-0.47, -0.21]$	-5.20	< .001
Age	0.01	0.00	[0.00, 0.01]	4.55	< .001
Sex - Male/Female	0.12	0.04	[0.04, 0.19]	3.05	.002
Sex - Other/Female	0.22	0.36	$[-0.48, 0.93]$	0.62	.535
Risk Perception	0.03	0.02	$[-0.01, 0.07]$	1.50	.134
Narcissism Admiration	-0.01	0.03	$[-0.07, 0.04]$	-0.52	.600
Narcissism Rivalry	-0.10	0.03	$[-0.15, -0.04]$	-3.53	< .001
Collective Narcissism	0.03	0.02	$[-0.02, 0.08]$	1.12	.263
Conspiracy Beliefs	-0.19	0.02	$[-0.24, -0.15]$	-7.89	< .001
National Identification	0.13	0.02	[0.09, 0.18]	6.11	< .001
Moral Circle	0.03	0.02	$[-0.01, 0.06]$	1.43	.154
Moral Identity Internalization	0.34	0.02	[0.29, 0.38]	14.02	< .001
Moral Identity Symbolization	0.05	0.02	[0.01, 0.10]	2.37	.018
Political Ideology	-0.02	0.02	$[-0.06, 0.02]$	-0.85	.394
Moral Circle × Internalization	-0.02	0.02	$[-0.06, 0.02]$	-0.92	.357
Moral Circle × Symbolization	0.04	0.02	[0.01, 0.08]	2.44	.015
Internalization × Political Ideology	-0.05	0.02	$[-0.09, -0.01]$	-2.71	.007
Symbolization × Political Ideology	0.06	0.02	[0.02, 0.09]	3.35	.001

Note: Internalization = Moral Identity Internalization; Symbolization = Moral Identity Symbolization

Table S12

Model 6: Full model predicting adherence to Physical Distancing measures (with Country included as a variable); R^2 = 0.42, *S.E.* = 0.77, adjusted R^2 = 0.41, F(35, 1914) = 38.94, p < .001.

	$\boldsymbol{b}$	***S.E.***	**95% CI**	$\boldsymbol{t(1914)}$	$\boldsymbol{p}$
Intercept	-0.53	0.08	[−0.68, −0.38]	-6.89	< .001
Country	0.51	0.14	[0.24, 0.77]	3.75	< .001
Age	0.01	0.00	[0.00, 0.01]	4.61	< .001
Sex - Male/Female	0.13	0.04	[0.05, 0.22]	3.14	.002
Sex - Other/Female	0.28	0.39	[−0.48, 1.04]	0.72	.472
Risk Perception	0.11	0.02	[0.06, 0.15]	4.62	< .001
Narcissism Admiration	0.01	0.03	[−0.05, 0.07]	0.24	.807
Narcissism Rivalry	-0.11	0.03	[−0.17, −0.05]	-3.62	< .001
Collective Narcissism	-0.02	0.03	[−0.07, 0.04]	-0.55	.585
Conspiracy Beliefs	-0.17	0.03	[−0.23, −0.12]	-6.09	< .001
National Identification	0.15	0.03	[0.10, 0.20]	5.84	< .001
Moral Circle	0.04	0.02	[0.00, 0.08]	1.91	.056
Moral Identity Internalization	0.35	0.03	[0.30, 0.41]	13.09	< .001
Moral Identity Symbolization	0.10	0.03	[0.05, 0.15]	4.12	< .001
Political Ideology	-0.02	0.02	[−0.07, 0.03]	-0.90	.371
Moral Circle × Internalization	-0.01	0.02	[−0.05, 0.03]	-0.49	.625
Moral Circle × Symbolization	0.04	0.02	[0.00, 0.08]	2.10	.036
Internalization × Political Ideology	-0.05	0.02	[−0.09, −0.01]	-2.67	.008
Symbolization × Political Ideology	0.07	0.02	[0.04, 0.11]	3.98	< .001
Country × Age	0.00	0.00	[−0.01, 0.00]	-0.60	.545
Country × Sex - Male/Female	0.02	0.09	[−0.15, 0.19]	0.23	.821
Country × Sex - Other/Female	0.52	0.88	[−1.21, 2.25]	0.59	.554
Country × Risk Perception	-0.15	0.05	[−0.25, −0.06]	-3.14	.002
Country × Narcissism Admiration	0.00	0.06	[−0.12, 0.12]	0.01	.990
Country × Narcissism Rivalry	-0.06	0.07	[−0.19, 0.07]	-0.87	.383
Country × Collective Narcissism	0.06	0.06	[−0.05, 0.16]	1.00	.318
Country × Conspiracy Beliefs	0.05	0.05	[−0.05, 0.16]	0.95	.343
Country × National Identification	-0.06	0.05	[−0.14, 0.03]	-1.23	.220
Country × Moral Circle	-0.05	0.04	[−0.14, 0.03]	-1.21	.225
Country × Internalization	-0.08	0.05	[−0.19, 0.02]	-1.52	.128
Country × Symbolization	-0.18	0.05	[−0.28, −0.07]	-3.35	.001
Country × Political Ideology	0.08	0.05	[−0.02, 0.19]	1.56	.119
Country × M. Circle × Internalization	-0.02	0.04	[−0.10, 0.07]	-0.38	.708
Country × M. Circle × Symbolization	-0.03	0.04	[−0.12, 0.05]	-0.78	.436
Country × Internalization × P. Ideology	0.11	0.05	[0.01, 0.21]	2.06	.039
Country × Symbolization × P. Ideology	-0.06	0.05	[−0.15, 0.03]	-1.26	.209

Note: Internalization = Moral Identity Internalization; Symbolization = Moral Identity Symbolization; M. Circle = Moral Circle; P. Ideology = Political Ideology

Figure S6

The relationship between Political Ideology and Internalization in predicting Physical Distancing for the total sample.

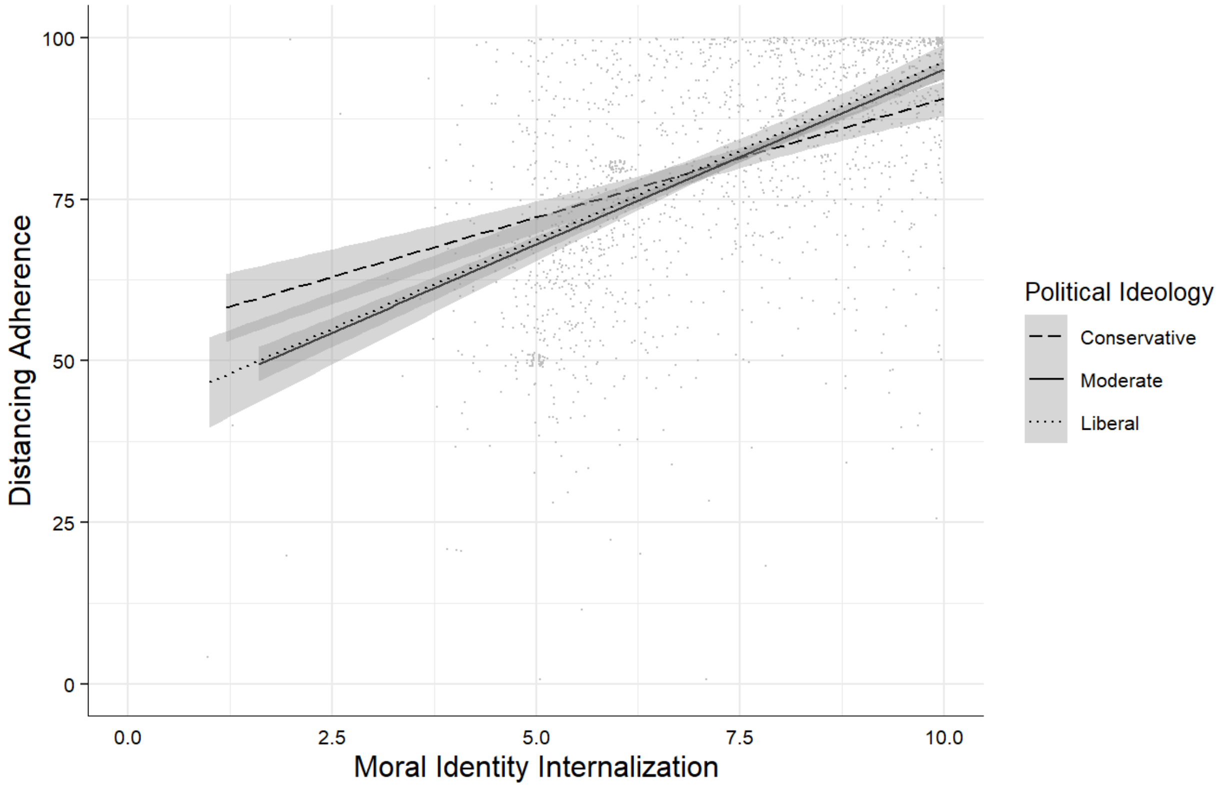

Figure S7

The relationship between Political Ideology and Internalization in predicting Physical Distancing for the total sample.

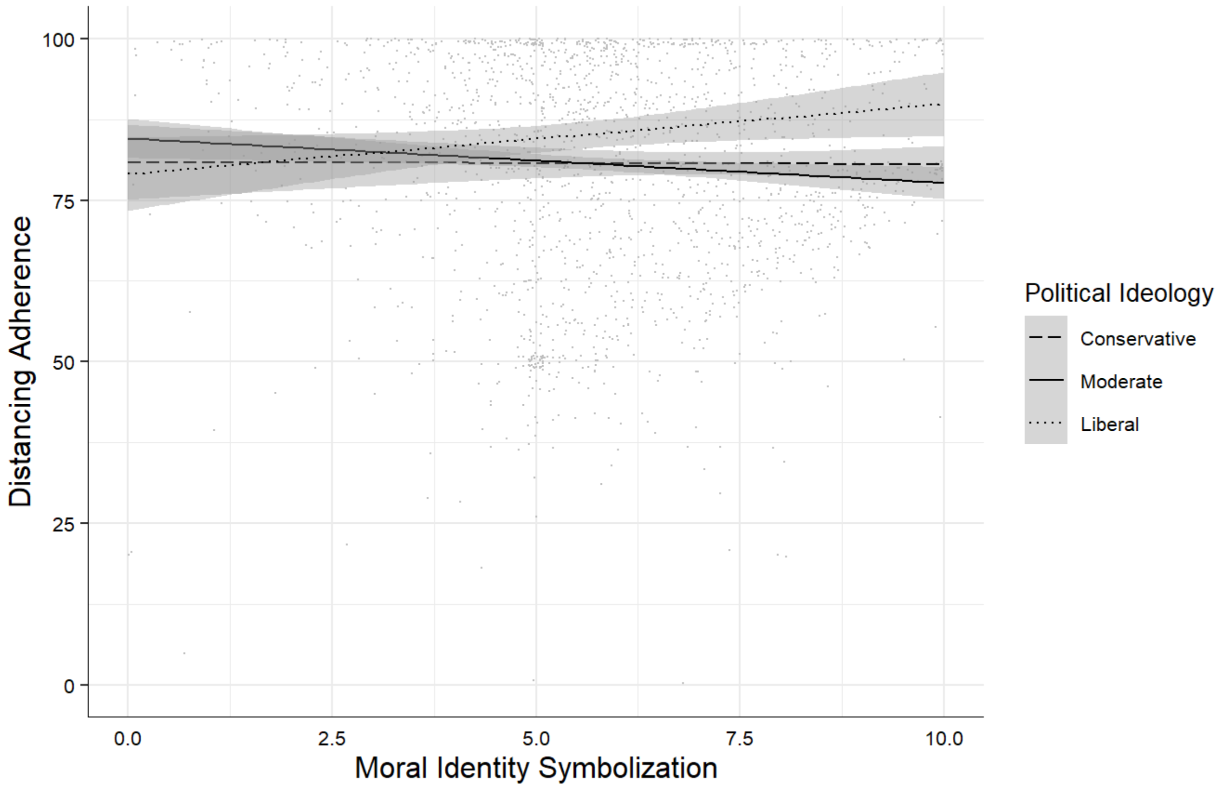

Table S13

Model 7: Predictors of Adherence to Physical Distancing measures in USA subsample; $R^2 = 0.4$, *S.E.* = 0.8, adjusted $R^2 = 0.39$, $F(17, 1430) = 55.3$, $p < .001$.

	b	*S.E.*	95% CI	$t(1430)$	p
Intercept	-0.53	0.08	[−0.68, −0.37]	-6.66	< .001
Age	0.01	0.00	[0.00, 0.01]	4.46	< .001
Sex - Male/Female	0.13	0.04	[0.05, 0.22]	3.03	.002
Sex - Other/Female	0.28	0.40	[−0.51, 1.07]	0.70	.486
Risk Perception	0.11	0.02	[0.06, 0.15]	4.47	< .001
Narcissism Admiration	0.01	0.03	[−0.05, 0.07]	0.24	.813
Narcissism Rivalry	-0.11	0.03	[−0.17, −0.05]	-3.50	< .001
Collective Narcissism	-0.02	0.03	[−0.07, 0.04]	-0.53	.598
Conspiracy Beliefs	-0.17	0.03	[−0.23, −0.11]	-5.89	< .001
National Identification	0.15	0.03	[0.10, 0.20]	5.64	< .001
Moral Circle	0.04	0.02	[0.00, 0.08]	1.85	.065
Moral Identity Internalization	0.35	0.03	[0.30, 0.41]	12.66	< .001
Moral Identity Symbolization	0.10	0.03	[0.05, 0.16]	3.99	< .001
Political Ideology	-0.02	0.03	[−0.07, 0.03]	-0.87	.387
Moral Circle × Internalization	-0.01	0.02	[−0.05, 0.03]	-0.47	.636
Moral Circle × Symbolization	0.04	0.02	[0.00, 0.08]	2.03	.043
Internalization × Political Ideology	-0.05	0.02	[−0.09, −0.01]	-2.59	.010
Symbolization × Political Ideology	0.07	0.02	[0.04, 0.11]	3.85	< .001

Note: Internalization = Moral Identity Internalization; Symbolization = Moral Identity Symbolization

Table S14

Model 8: Predictors of Adherence to Physical Distancing measures in New Zealand subsample;

R^2 = 0.32, *S.E.* = 0.69, adjusted R^2 = 0.3, F(17, 484) = 13.4, p < .001.

	b	***S.E.***	**95% CI**	***t*(484)**	***p***
Intercept	-0.02	0.10	[−0.22, 0.18]	-0.21	.837
Age	0.01	0.00	[0.00, 0.01]	2.72	.007
Sex - Male/Female	0.15	0.07	[0.02, 0.28]	2.33	.020
Sex - Other/Female	0.80	0.71	[−0.58, 2.19]	1.14	.256
Risk Perception	-0.05	0.04	[−0.12, 0.03]	-1.22	.223
Narcissism Admiration	0.01	0.05	[−0.09, 0.10]	0.17	.867
Narcissism Rivalry	-0.17	0.05	[−0.27, −0.06]	-3.21	.001
Collective Narcissism	0.04	0.04	[−0.04, 0.12]	0.94	.349
Conspiracy Beliefs	-0.12	0.04	[−0.20, −0.04]	-2.96	.003
National Identification	0.09	0.03	[0.03, 0.16]	2.81	.005
Moral Circle	-0.01	0.03	[−0.08, 0.05]	-0.33	.741
Moral Identity Internalization	0.27	0.04	[0.19, 0.35]	6.32	< .001
Moral Identity Symbolization	-0.07	0.04	[−0.15, 0.01]	-1.73	.085
Political Ideology	0.06	0.04	[−0.02, 0.14]	1.45	.148
Moral Circle × Internalization	-0.03	0.04	[−0.10, 0.04]	-0.77	.444
Moral Circle × Symbolization	0.01	0.03	[−0.06, 0.07]	0.21	.831
Internalization × Political Ideology	0.05	0.04	[−0.03, 0.14]	1.25	.213
Symbolization × Political Ideology	0.01	0.04	[−0.06, 0.09]	0.35	.726

Note: Internalization = Moral Identity Internalization; Symbolization = Moral Identity Symbolization

Hygiene Adherence

Table S15

Model 9: Full regression model predicting adherence to Physical Hygiene measures (without Country); R^2 = 0.27, *S.E.* = 0.86, adjusted R^2 = 0.26, F(17, 1931) = 41.61, p < .001.

predictor	***b***	***S.E.***	**95% CI**	$t(1931)$	***p***
Intercept	-0.22	0.07	[−0.36, −0.09]	-3.24	.001
Age	0.00	0.00	[0.00, 0.01]	1.82	.069
Sex - Male/Female	0.19	0.04	[0.11, 0.27]	4.56	< .001
Sex - Other/Female	-0.47	0.39	[−1.23, 0.28]	-1.22	.221
Risk Perception	0.13	0.02	[0.08, 0.17]	5.78	< .001
Narcissism Admiration	0.10	0.03	[0.05, 0.16]	3.66	< .001
Narcissism Rivalry	-0.12	0.03	[−0.18, −0.06]	-4.00	< .001
Collective Narcissism	0.14	0.03	[0.09, 0.19]	5.19	< .001
Conspiracy Beliefs	-0.08	0.03	[−0.13, −0.02]	-2.89	.004
National Identification	0.13	0.02	[0.09, 0.18]	5.73	< .001
Moral Circle	0.01	0.02	[−0.03, 0.05]	0.67	.505
Moral Identity Internalization	0.20	0.03	[0.15, 0.25]	7.82	< .001
Moral Identity Symbolization	0.23	0.02	[0.18, 0.28]	9.44	< .001
Political Ideology	0.02	0.02	[−0.02, 0.07]	1.04	.300
Moral Circle × Internalization	0.02	0.02	[−0.02, 0.06]	1.02	.306
Moral Circle × Symbolization	0.01	0.02	[−0.03, 0.05]	0.50	.618
Internalization × Political Ideology	-0.06	0.02	[−0.10, −0.02]	-3.17	.002
Symbolization × Political Ideology	0.03	0.02	[−0.01, 0.07]	1.68	.094

Note: Internalization = Moral Identity Internalization; Symbolization = Moral Identity Symbolization

Table S16

Model 10: Full model predicting adherence to Physical Hygiene measures (with Country included as a variable); R^2 = 0.28, *S.E.* = 0.85, adjusted R^2 = 0.27, $F(35, 1913) = 21.76$, $p < .001$.

	b	***S.E.***	**95% CI**	$t(1913)$	***p***
Intercept	-0.25	0.12	[−0.49, 0.00]	-1.99	.046
Country	0.02	0.15	[−0.27, 0.32]	0.17	.869
Age	0.00	0.00	[−0.01, 0.00]	-0.47	.640
Sex - Male/Female	0.22	0.08	[0.06, 0.38]	2.71	.007
Sex - Other/Female	-0.22	0.87	[−1.93, 1.50]	-0.25	.804
Risk Perception	-0.04	0.05	[−0.13, 0.06]	-0.76	.446
Narcissism Admiration	0.06	0.06	[−0.05, 0.18]	1.08	.279
Narcissism Rivalry	-0.12	0.06	[−0.25, 0.01]	-1.88	.060
Collective Narcissism	0.15	0.05	[0.05, 0.25]	2.92	.004
Conspiracy Beliefs	-0.01	0.05	[−0.11, 0.09]	-0.17	.861
National Identification	0.06	0.04	[−0.02, 0.14]	1.52	.128
Moral Circle	0.01	0.04	[−0.07, 0.09]	0.15	.879
Moral Identity Internalization	0.09	0.05	[−0.01, 0.20]	1.79	.074
Moral Identity Symbolization	0.22	0.05	[0.12, 0.32]	4.37	< .001
Political Ideology	0.02	0.05	[−0.08, 0.12]	0.36	.719
Moral Circle × Internalization	0.00	0.04	[−0.09, 0.08]	-0.01	.991
Moral Circle × Symbolization	-0.07	0.04	[−0.15, 0.02]	-1.58	.114
Internalization × Political Ideology	-0.12	0.05	[−0.22, −0.02]	-2.28	.023
Symbolization × Political Ideology	-0.01	0.05	[−0.10, 0.09]	-0.11	.914
Country × Age	0.00	0.00	[0.00, 0.01]	1.50	.133
Country × Sex - Male/Female	-0.03	0.09	[−0.22, 0.15]	-0.33	.739
Country × Sex - Other/Female	-0.39	0.97	[−2.30, 1.52]	-0.40	.688
Country × Risk perception	0.18	0.05	[0.07, 0.28]	3.28	.001
Country × Narcissism admiration	0.04	0.07	[−0.09, 0.17]	0.60	.547
Country × Narcissism rivalry	0.01	0.07	[−0.13, 0.15]	0.16	.871
Country × Collective narcissism	-0.01	0.06	[−0.13, 0.11]	-0.12	.901
Country × Conspiracy	-0.09	0.06	[−0.20, 0.03]	-1.44	.151
Country × National Identification	0.10	0.05	[0.00, 0.20]	1.98	.048
Country × Moral Circle	0.00	0.05	[−0.09, 0.09]	0.04	.965
Country × Internalization	0.13	0.06	[0.01, 0.25]	2.09	.037
Country × Symbolization	-0.01	0.06	[−0.12, 0.11]	-0.10	.923
Country × Political Ideology	-0.01	0.06	[−0.12, 0.11]	-0.14	.891
Country × M. Circle × Internalization	0.03	0.05	[−0.07, 0.12]	0.52	.602
Country × M. Circle × Symbolization	0.09	0.05	[0.00, 0.18]	1.89	.058
Country × Internalization × P. Ideology	0.06	0.06	[−0.06, 0.17]	0.98	.327
Country × Symbolization × P. Ideology	0.04	0.05	[−0.06, 0.14]	0.71	.476

Note: Internalization = Moral Identity Internalization; Symbolization = Moral Identity Symbolization; M. Circle = Moral Circle; P. Ideology = Political Ideology

Figure S8

The relationship between Political Ideology and Internalization predicting Physical Hygiene for the total sample.

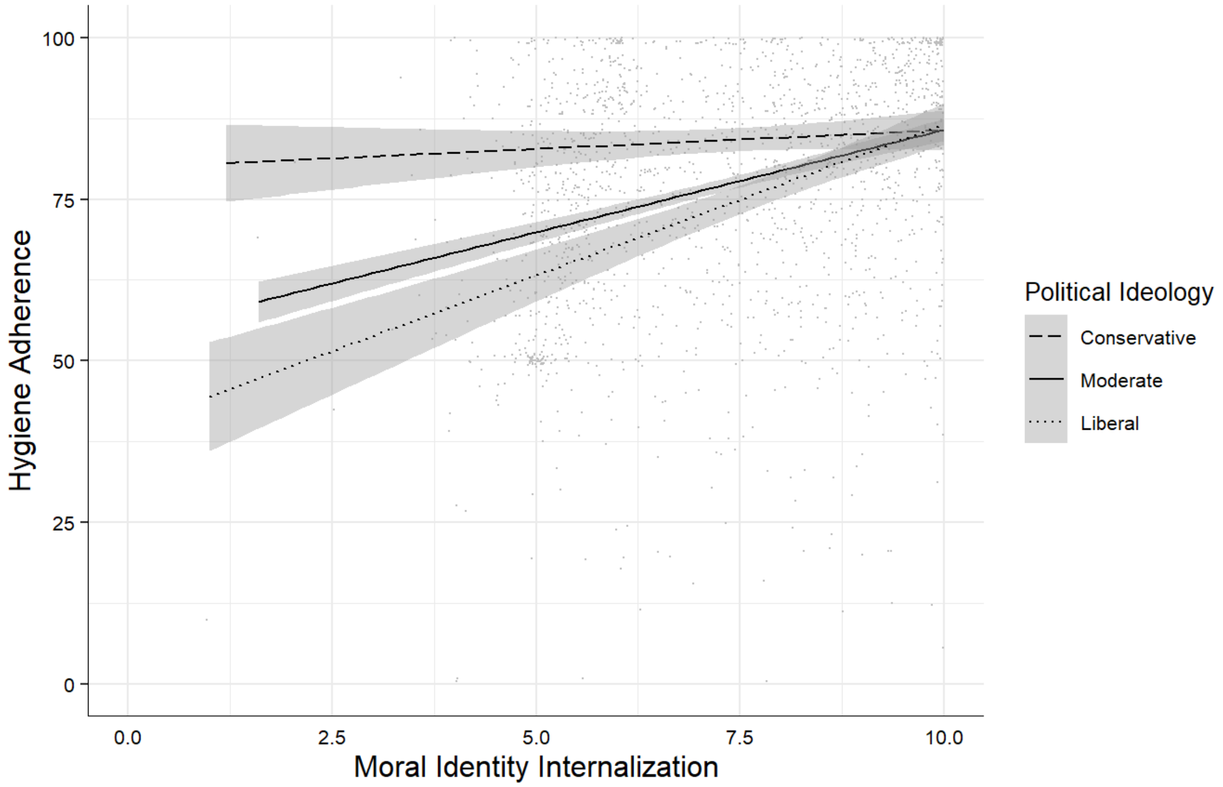

Figure S9

The relationship between Political Ideology and Moral Identity Symbolization in predicting Physical Hygiene for the total sample.

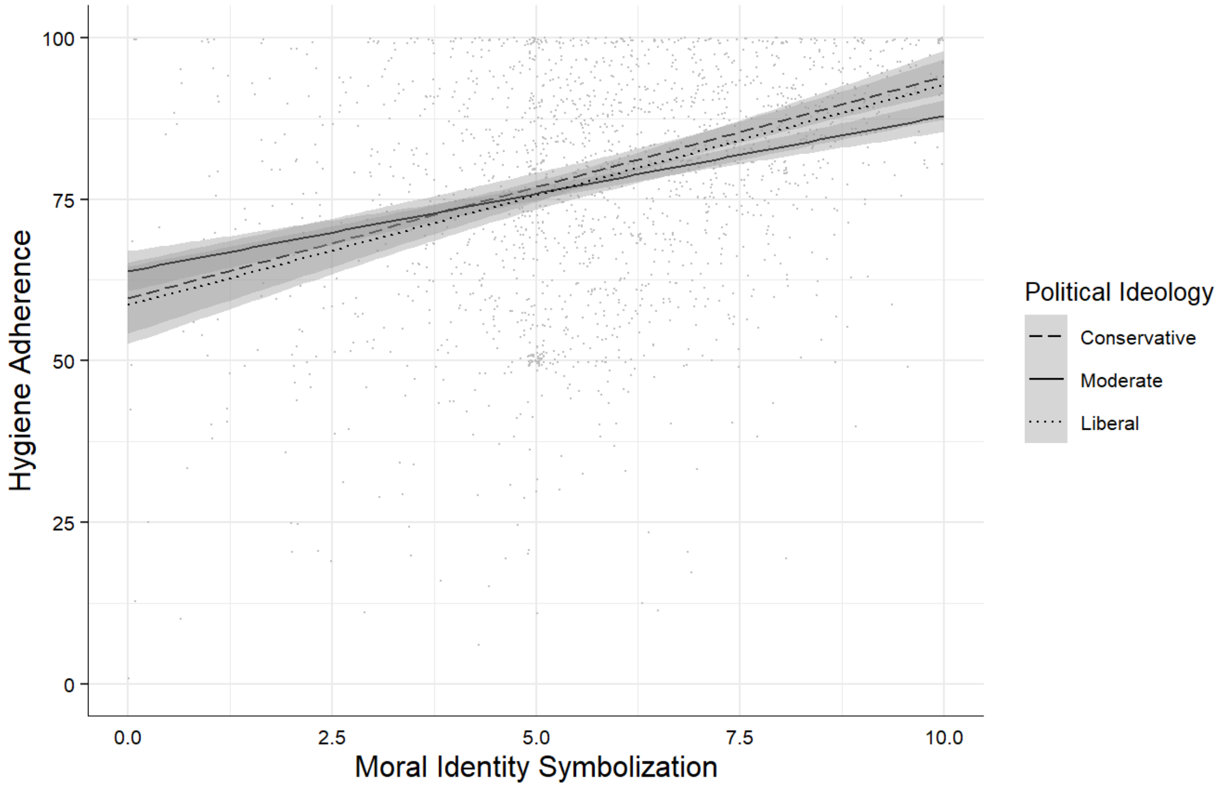

Table S17

Model 11: Predictors of Adherence to Physical Hygiene measures in USA subsample; R^2 = 0.31, *S.E.* = 0.83, adjusted R^2 = 0.3, $F(17, 1429) = 37.46$, $p < .001$.

	b	*S.E.*	95% CI	$t(1429)$	p
Intercept	-0.22	0.08	[−0.38, −0.06]	-2.67	.008
Age	0.00	0.00	[0.00, 0.01]	2.02	.044
Sex - Male/Female	0.19	0.05	[0.10, 0.28]	4.10	< .001
Sex - Other/Female	-0.61	0.42	[−1.43, 0.22]	-1.45	.148
Risk Perception	0.14	0.02	[0.09, 0.19]	5.63	< .001
Narcissism Admiration	0.10	0.03	[0.04, 0.17]	3.28	.001
Narcissism Rivalry	-0.11	0.03	[−0.17, −0.05]	-3.34	.001
Collective Narcissism	0.14	0.03	[0.08, 0.21]	4.64	< .001
Conspiracy Beliefs	-0.09	0.03	[−0.15, −0.03]	-3.09	.002
National Identification	0.16	0.03	[0.11, 0.22]	5.85	< .001
Moral Circle	0.01	0.02	[−0.04, 0.05]	0.36	.721
Moral Identity Internalization	0.22	0.03	[0.16, 0.28]	7.58	< .001
Moral Identity Symbolization	0.22	0.03	[0.16, 0.27]	7.84	< .001
Political Ideology	0.01	0.03	[−0.04, 0.06]	0.41	.685
Moral Circle × Internalization	0.03	0.02	[−0.02, 0.07]	1.12	.264
Moral Circle × Symbolization	0.02	0.02	[−0.02, 0.06]	1.09	.276
Internalization × Political Ideology	-0.06	0.02	[−0.11, −0.02]	-2.95	.003
Symbolization × Political Ideology	0.03	0.02	[−0.01, 0.07]	1.62	.106

Note: Internalization = Moral Identity Internalization; Symbolization = Moral Identity Symbolization

Table S18

Model 12: Predictors of Adherence to Physical Hygiene measures in New Zealand subsample; R^2 = 0.17, *S.E.* = 0.9, adjusted R^2 = 0.14, $F(17, 484) = 5.93$, $p < .001$.

	b	***S.E.***	**95% CI**	$t(484)$	***p***
Intercept	-0.25	0.13	[−0.50, 0.01]	-1.88	.061
Age	0.00	0.00	[−0.01, 0.00]	-0.44	.660
Sex - Male/Female	0.22	0.09	[0.05, 0.39]	2.55	.011
Sex - Other/Female	-0.22	0.93	[−2.04, 1.60]	-0.23	.815
Risk Perception	-0.04	0.05	[−0.14, 0.06]	-0.72	.472
Narcissism Admiration	0.06	0.06	[−0.06, 0.19]	1.02	.308
Narcissism Rivalry	-0.12	0.07	[−0.26, 0.01]	-1.77	.077
Collective Narcissism	0.15	0.06	[0.04, 0.26]	2.75	.006
Conspiracy Beliefs	-0.01	0.05	[−0.11, 0.10]	-0.16	.869
National Identification	0.06	0.04	[−0.02, 0.15]	1.43	.152
Moral Circle	0.01	0.04	[−0.08, 0.09]	0.14	.886
Moral Identity Internalization	0.09	0.06	[−0.02, 0.20]	1.68	.093
Moral Identity Symbolization	0.22	0.05	[0.12, 0.33]	4.12	< .001
Political Ideology	0.02	0.06	[−0.09, 0.13]	0.34	.734
Moral Circle × Internalization	0.00	0.05	[−0.09, 0.09]	-0.01	.992
Moral Circle × Symbolization	-0.07	0.04	[−0.15, 0.02]	-1.49	.137
Internalization × Political Ideology	-0.12	0.06	[−0.23, −0.01]	-2.15	.032
Symbolization × Political Ideology	-0.01	0.05	[−0.10, 0.09]	-0.10	.919

Note: Internalization = Moral Identity Internalization; Symbolization = Moral Identity Symbolization

Table S19

Summary of support for hypotheses for each measure

	Predictor	Restrictions Support	Distancing Adherence	Hygiene Adherence
H1a	Internalization	✓	✓	✓
	USA: internalization	✓	✓	✓
	NZL: internalization	✓	✓	✗
H1b	Symbolization	✓	✓	✓
	USA: symbolization	✓	✓	✓
	NZL: symbolization	✗	✗	✓
H2a	Country × internalization × political ideology	✓	✓	✗
H2b	Country × symbolization × political ideology	✗	✗	✗
H3a	USA: internalization × political ideology	✓	✓	✓
H3b	USA: symbolization × political ideology	✓	✓	✗
H4a	NZL: no internalization × political ideology	✓	✓	✗
H4b	NZL: no symbolization × political ideology	✓	✓	✓

Analysis of Opposition to Mitigation Measures

Restrictions Support

A multinomial logistic regression was conducted to test the predictors of opposition to restrictions. Overall, the model was significant, $\chi^2(6, N = 1971) = 132.51$, $p < .001$, and explained between 6.52% (Cox and Snell R^2) and 13.9% (Nadelkerke R^2) of the variance in responses.

Table S20

Predictors of opposition to restrictions

	B	***SE***	**Wald**	***p***	***OR***	**2.5%**	**97.5%**
(Intercept)	1.66	0.92	1.81	.070	5.28	0.90	33.07
Country: USA	1.24	0.26	4.84	< .001**	3.45	2.14	5.86
Internalization	-0.07	0.01	-5.73	< .001**	0.93	0.91	0.96
Symbolization	-0.02	0.01	-1.74	.081	0.98	0.96	1.00
Political Ideology	-0.38	0.13	-2.91	.004*	0.68	0.53	0.88
Internalization × Ideology	0.01	0.00	5.12	< .001**	1.01	1.01	1.01
Symbolization × Ideology	0.00	0.00	-0.95	.340	1.00	1.00	1.00

Note. * = sig. at $p < .05$; ** = sig. at $p < .001$

Figure S10

Interaction between Political Ideology and Moral Identity Internalization in predicting opposition to restrictions

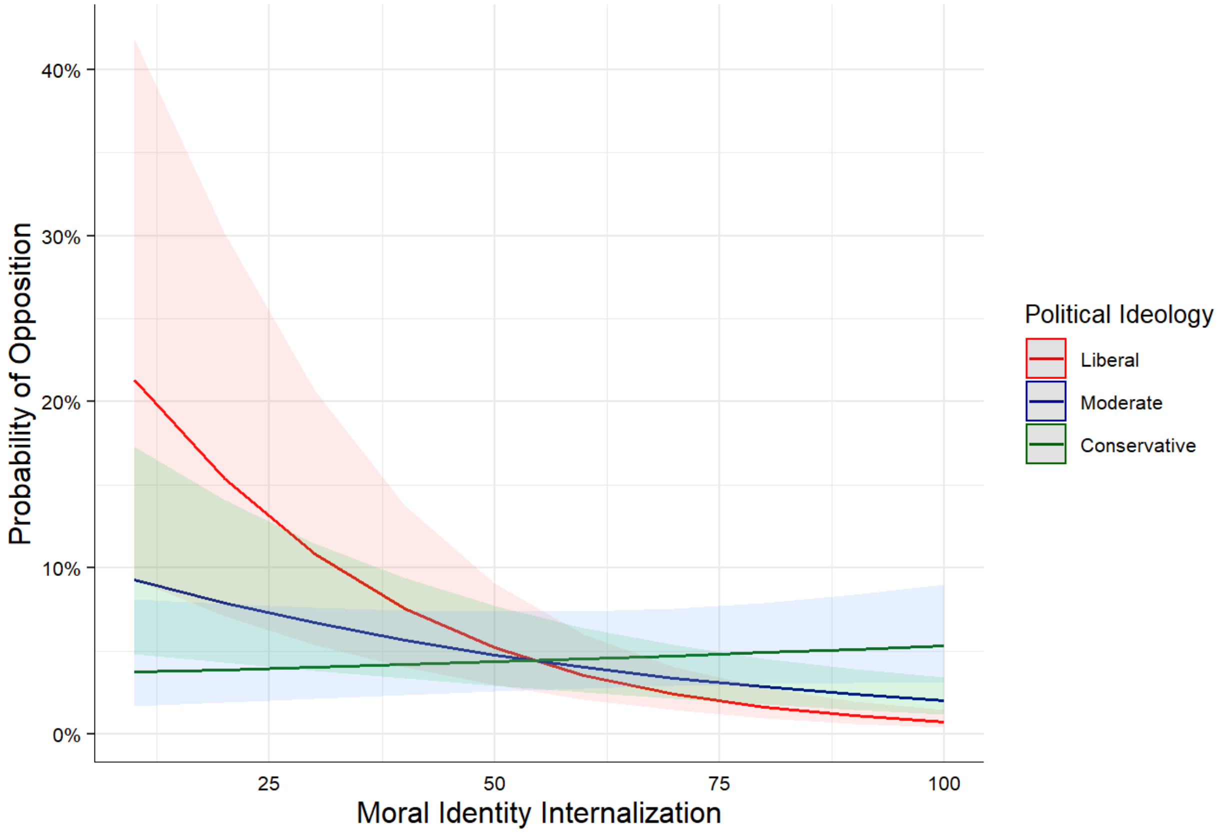

Physical Contact Measures

A multinomial logistic regression was conducted to test the predictors of opposition to physical contact measures. Overall the model was significant, $\chi^2(6, N = 1971) = 78.19$, $p < .001$, and explained between 3.9% (Cox and Snell R^2) and 13.11% (Nadelkerke R^2) of the variance in responses.

Table S21

Predictors of opposition to physical contact measures

	B	***SE***	**Wald**	***p***	***OR***	**2.5%**	**97.5%**
(Intercept)	1.07	1.09	0.98	.327	2.92	0.35	26.41
Country: USA	1.07	0.36	2.94	.003*	2.92	1.50	6.38
Internalization	-0.07	0.02	-4.32	< .001**	0.93	0.90	0.96
Symbolization	0.00	0.01	-0.28	.778	1.00	0.97	1.02
Political Ideology	-0.20	0.16	-1.20	.229	0.82	0.59	1.13
Internalization × Ideology	0.00	0.00	1.87	.062	1.00	1.00	1.01
Symbolization × Ideology	0.00	0.00	-1.06	.291	1.00	0.99	1.00

Note. * = sig. at $p < .05$; ** = sig. at $p < .001$

Physical Hygiene Measures

A multinomial logistic regression was conducted to test the predictors of opposition to physical hygiene measures. Overall the model was significant, $\chi^2(6, N = 1971) = 98.85$, $p < .001$, and explained between 4.91% (Cox and Snell R^2) and 13.85% (Nadelkerke R^2) of the variance in responses.

Table S22

Predictors of opposition to physical hygiene measures

	B	***SE***	**Wald**	***p***	***OR***	**2.5%**	**97.5%**
(Intercept)	2.36	0.96	2.45	.014*	10.58	1.66	74.65
Country: USA	-0.21	0.22	-0.94	.348	0.81	0.53	1.26
Internalization	-0.05	0.01	-3.81	< .001**	0.95	0.93	0.98
Symbolization	-0.03	0.01	-2.79	.005*	0.97	0.95	0.99
Political Ideology	-0.29	0.16	-1.88	.061	0.75	0.55	1.01
Internalization × Ideology	0.01	0.00	2.64	.008*	1.01	1.00	1.01
Symbolization × Ideology	0.00	0.00	-0.75	.451	1.00	1.00	1.00

Note. * = sig. at $p < .05$; ** = sig. at $p < .001$

Figure S11

Interaction between Political Ideology and Moral Identity Internalization in predicting opposition to hygiene measures

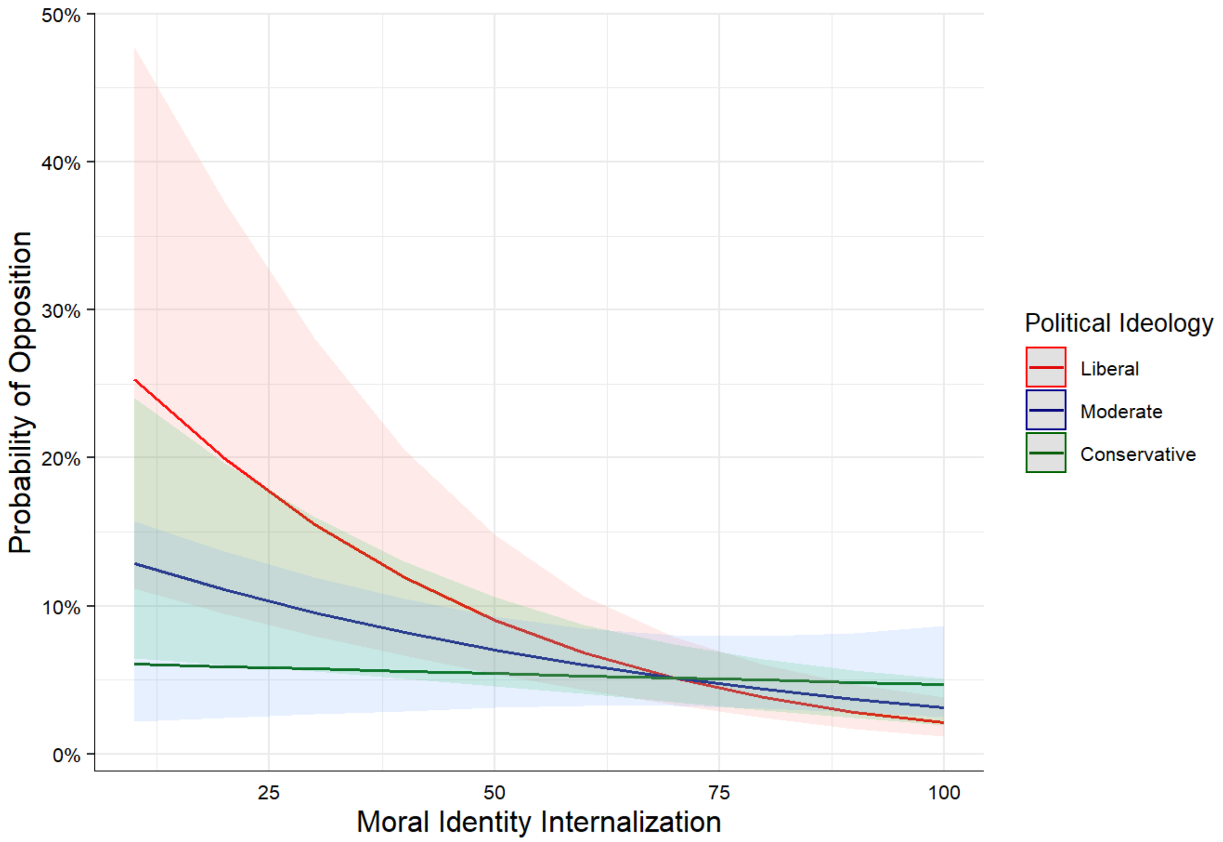

Supplement: Supplementary file 1 — Figure S1. Residuals by country, predicting restrictions support. Figure S2. Residuals by country, predicting adherence to physical distancing measures. Figure S3. Residuals by country, predicting adherence to physical hygiene measures. Table S1. Participant Characteristics (N = 1,980) Table S2. Correlations Between All Variables Including Covariates for the Total Sample Table S3. Correlations Between All Variables Including Covariates for the USA Subsample Table S4. Correlations Between All Variables Including Covariates for the New Zealand Subsample Table S5. Means, Standard Deviations, and Differences Between the Subsamples Table S6. Means, Standard Deviations, and Differences Depending on Gender Table S7. Model 1: Full Regression Model Predicting Restrictions Support (Without Country Table S8. Model 2: Full Regression Model Predicting Restrictions Support (With Country Included as a Variable) Figure S4. The relationship between political ideology and internalization in predicting restrictions support for the total sample. Figure S5. The relationship between political ideology and symbolization in predicting restrictions support for the total sample. Table S9. Model 3: Predictors of Restrictions Support in USA Subsample Table S10. Model 4: Predictors of Restrictions Support in New Zealand Subsample Table S11. Model 5: Full Regression Model Predicting Adherence to Physical Distancing Measures (Without Country) Table S12. Model 6: Full Model Predicting Adherence to Physical Distancing Measures (With Country Included as a Variable) Figure S6. The relationship between political ideology and internalization in predicting physical distancing for the total sample. Figure S7. The relationship between political ideology and symbolization in predicting physical distancing for the total sample. Table S13. Model 7: Predictors of Adherence to Physical Distancing Measures in the USA Subsample Table S14. Model 8: Predictors of Adherence to Physical Distancing Measures in the Ne [file POPS-9999-0-s001.pdf]
